# Ranking Candidate Disease Genes from Gene Expression and Protein Interaction: A Katz-Centrality Based Approach

**DOI:** 10.1371/journal.pone.0024306

**Published:** 2011-09-02

**Authors:** Jing Zhao, Ting-Hong Yang, Yongxu Huang, Petter Holme

**Affiliations:** 1 Department of Mathematics, Logistical Engineering University, Chongqing, China; 2 Department of Health Policy & Management, University of Pittsburgh, Pittsburgh, Pennsylvania, United States of America; 3 IceLab, Department of Physics, Umeå University, Umeå, Sweden; 4 Department of Energy Science, Sungkyunkwan University, Suwon, Korea; 5 Kavli Institute for Theoretical Physics China (KITPC), Chinese Academy of Sciences, Beijing, China; University of Maribor, Slovenia

## Abstract

Many diseases have complex genetic causes, where a set of alleles can affect the propensity of getting the disease. The identification of such disease genes is important to understand the mechanistic and evolutionary aspects of pathogenesis, improve diagnosis and treatment of the disease, and aid in drug discovery. Current genetic studies typically identify chromosomal regions associated specific diseases. But picking out an unknown disease gene from hundreds of candidates located on the same genomic interval is still challenging. In this study, we propose an approach to prioritize candidate genes by integrating data of gene expression level, protein-protein interaction strength and known disease genes. Our method is based only on two, simple, biologically motivated assumptions—that a gene is a good disease-gene candidate if it is differentially expressed in cases and controls, or that it is close to other disease-gene candidates in its protein interaction network. We tested our method on 40 diseases in 58 gene expression datasets of the NCBI Gene Expression Omnibus database. On these datasets our method is able to predict unknown disease genes as well as identifying pleiotropic genes involved in the physiological cellular processes of many diseases. Our study not only provides an effective algorithm for prioritizing candidate disease genes but is also a way to discover phenotypic interdependency, cooccurrence and shared pathophysiology between different disorders.

## Introduction

Many diseases need complex genetic and environmental factors to occur. To find the genetic factors is important for both medical (aiding in drug discovery and personalized treatments) and scientific reasons (understanding mechanistic and evolutionary aspects of pathogenesis). Genetic approaches, such as linkage analysis (connecting loci with a tendency to be inherited together) and association studies (mapping correlation between alleles at different loci), have uncovered plenty of links between diseases and particular chromosomal regions [1]. In such studies, a chromosomal region typically contains up to hundreds of genes, which is too much to be useful to experimentally test potential disease genes. For this reason it is very valuable with computational methods to rank such candidate genes within a chromosomal region in order of likeliness of being a disease gene.

It is fairly well confirmed that the propensity of many diseases can be reflected in a difference of gene expression levels in particular cell types [2]. Specifically, if a group of genes shows a consistent pattern of different expression levels in sick subjects and a control group, then that gene is likely a strong candidate of playing a pathogenic role. Differences in expression levels are detected primarily by microarray studies [2]–[6]. Another phenomenon pointed out by previous studies [7]–[9] is that genes associated with the same disorder tend to share common functional features, reflected in that their protein products have a tendency to interact with each other. Thus another indicative trait of a disease gene is that its protein product is strongly linked to other disease-gene proteins. A few previous computational methods have taken this starting point and devised methods to identify disease genes from protein-protein interactions [10]–[13]. Recently, some efforts have been made to integrate these different contributions—being differentially expressed and being close to diseases genes, for the identification of disease genes [14], [15]. This category of methods is based on the assumption that the protein products of disease genes tend to be in close, in the protein interaction network, to differentially expressed genes. Karni *et al.* noticed that this problem as one equivalent to the set cover problem in graph theory, which is NP-complete [14]. Thus it is no wonder that large-scale protein networks can only be analyzed with approximate, greedy algorithms. Nitsch *et al.* defined, what they call, a soft neighborhood of differentially expressed genes where indirectly connected genes also can contribute but with a weight decreasing with the distance [15]. Our method is similar in that it combines the same types of data, but rather than assuming that nodes neighboring to differentially expressed genes are disease gene candidates, we assume, recursively, that nodes close to disease gene candidates are disease gene candidates. This difference, as we will see, simplifies our method both conceptually and algorithmically, and makes it to a better tool for inferring pathogenic interactions invisible in microarray data.

To outline the paper, we will start by deriving out method from our simple assumptions of influence inspired by the Katz centrality [16], which is similar in nature to the more well known PageRank algorithm. To test our method, we apply it to 58 gene expression datasets from major platforms in the NCBI Gene Expression Omnibus (GEO) database. These datasets represent the gene expression levels of 40 distinct diseases. Our human protein interaction data comes from the STRING database of the human genome and proteome. We got the data on disease genes of the mentioned 40 diseases from the OMIM database. First, we predicted disease genes within disease-associated loci only based on gene expression levels and protein-protein interactions. We used known disease genes as a benchmark to test the performance. Then we demonstrated that inputting known disease genes enhanced the prediction accuracy. At last, we analyzed the globally top ranked genes to confirm that they are involved in the physiological cellular processes of many diseases.

## Results and Discussion

### Overview and derivation of the method

In this section, we will derive our method for assigning a score to genes to reflect how strong candidate disease gene a node is. The derivation follows the same ideas as Katz' centrality index designed for social networks [16] and similar indices [17], [18]. The starting point from the derivation is the assumption that disease genes are typically close, in the associated protein network, to other disease genes [7]. This is natural since proteins typically need to form complexes, or in other ways interact to be involved in the same (pathogenic, in this case) function, hence their associated proteins should also have a tendency to interact. We let **s**  =  (*s*_1_,…,*s_n_*) be our score vector over the set of genes (where *s_i_* indicates how strong *i* is as a disease-gene candidate), and treat the score as a property that can be redistributed by the nodes, then our starting point can be formalized mathematically as
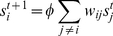
(1)where *w_ij_* is the strength of the coupling between the proteins of gene *i* and *j*, *φ* is a constant telling us how strongly *i* is affected by the scores of its neighborhood, and *t* (in the superscript) is a symbolic, discrete time of the redistribution of score (that we will get rid of eventually). However, in Eq. (1), we do not include the activity level of gene *i* in the disease, such as difference in expression level. We let **x**  =  (*x*_1_,…,*x_n_*)*^T^* represent activity level of genes in the disease, quantified in some way. Assuming that the *x_i_* influence the score of *i* in the same way as the score of the neighbors do, we can extend Eq. (1) to
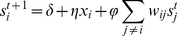
(2)where *η* is another proportionality constant. *δ* is a constant that represents a background probability that a vertex is a disease gene even though in is neither differentially expressed nor connected to other disease genes. The time in this equation is just symbolic, we are interested in the situation when all the scores are redistributed to a stationary state so **s** *^t^* ^+1^  =  **s** *^t^*. Then we drop the superscript and write Eq. (2) on matrix format as
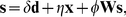
(3)where **d**  =  (1,…,1)*^T^*. Which gives
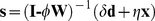
(4)Since we are only interested in the relative values of the scores we can set *δ = 1* without loss of generality. If we assume the activity levels are the same, i.e. **x**  =  **d**, and that the coupling strengths of **W** are one or zero, and that **d** is negligible (i.e. that *η* is large), then our score function reduces to the Katz centrality. The score function has two free parameters—*φ* that sets the balance between the influence of the neighbors in the protein network and the difference in activity level; and *η* that sets the relative likelihood that a random vertex is a candidate gene. If *φ* is small, the difference in activity level is more important; if *φ* is large, the coupling to the protein neighbors is more important. Another limit on *φ* is that the elements of (**I** – *φ* **W**)^−1^ should be non-negative, which in practice will be the case for the *φ* optimizing the score (and thus no practical problem). If *η* is small there is less value in the differential expression data so that there is a fair chance a random node is associated with the disease. Ultimately, one needs to calibrate *φ* and *η* with real data where one has another estimate of how much a gene contribute to the disease. We will do this below, but first we consider an example to illustrate the procedure.

**Figure 1:**
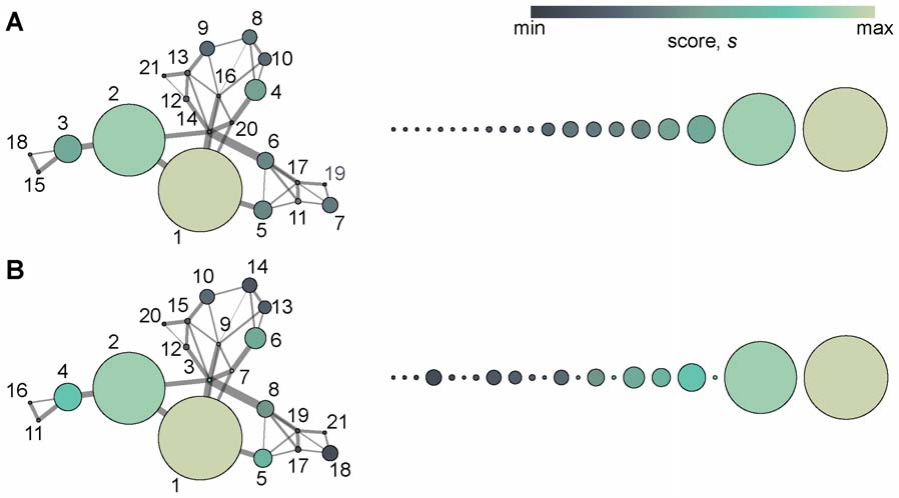
Illustration of the method with synthetic data. The area of the nodes is proportional to *x_i_*—the difference in expression level. The width of the edges represents the coupling strength *w_ij_* in the protein interaction network. The color of the nodes represents our score and the numbers shows their order in this ranking. Panels A and B shows the result of two values of *φ*—a low value of *φ* (2% of the maximal possible) in A, and a high value of *φ* (98% of max). Low *φ*-values put an emphasis on the difference in expression level; high *φ*-values stress the proximity to other vertices with high score. We also assume *η≫1*.

In Fig. 1 we illustrate the method on an example network designed to capture some features of disease gene networks. The area of a node *i* is proportional to *x_i_*; the width of an edge is proportional to the *w_ij_*; the color indicate the score *s_i_* and the number shows the ranking of the vertices. In this case we assume *η* ≫ 1 so that **d** and *η* can be omitted in Eq. (4). In panel A we show the situation for a low *φ*-value—about 2% of its maximum value (that comes from the condition that all elements of (**I** – *φ* **W**)^–1^ should be non-negative); in B we illustrate the opposite case of a large *φ* (98% of the maximum). We see that the *φ* puts a priority on being close to vertices of high score so that, for example, the vertex that is ranked 14th in A (that it self is not differentially expressed) becomes ranked third in B.

### Disease gene prediction based on gene expression levels and protein-protein interactions

We collected 58 human microarray datasets representing 40 diseases from the NCBI Gene Expression Omnibus (GEO). Since GEO contains some experiments that include gene expression measurements for more than one disease, we combined the samples of one disease and the normal samples in the same experiment into a disease-control set and generated 81 disease-control sets from the 58 datasets (see Table S1). Our protein interaction network was constructed from the STRING database, which includes both physical and functional interactions integrated from numerous sources, including experimental repositories, computational prediction methods and public text collections. Eliminating self-interactions, this network consists of 1,032,872 interactions between 14,532 proteins of human genome, with their normalized interaction weights in the STRING database.

For each microarray disease-control set, we calculated its *s*-core vector by equation (4). Here we set the vector **x** in Eq. (4) as the vector of the absolute values of the logarithm of the ratio of the expression levels of this microarray set, and **W** as the normalized interaction-weight matrix in STRING (See Material and Method). In the cases when one disease corresponds to more than one experiment (disease-control datasets) we summed up the *s*-score vectors corresponding to the disease. Then we ranked the genes in each candidate-gene set of a disease according to their *s*-scores and got their *r*-ratios (see the Materials and Methods section). We scanned the (*φ*,*η*) parameter space in the regions 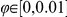 and 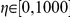 using this procedure, and checked the average *r*-ratios of all the known OMIM disease genes for the disease we studied. It is noted that, since some genes are involved in different diseases, for example, the gene IL6 is associated with Type 2 Diabetes Mellitus, Crohn's Disease and Juvenile Rheumatoid Arthritis, we actually computed 348 *r*-ratios for the known 318 distinct OMIM disease genes of the 40 diseases. In this way, the optimum value of (*φ*,*η*) was determined as (0.005,39), which minimized the average *r*-ratios of known OMIM disease genes for the 40 diseases. For comparison, we also fixed *φ* and *η* to zero respectively, and then searched for the optimum *η* and *φ* as above. They represent the cases that only expression data (*φ* = 0) and protein interaction network (*η* = 0) were used to predict disease genes, respectively. When *φ * =  0, the result is the same for any *η *>0. As for *η* = 0, we got optimum parameter *φ* = 0.001.

**Figure 2:**
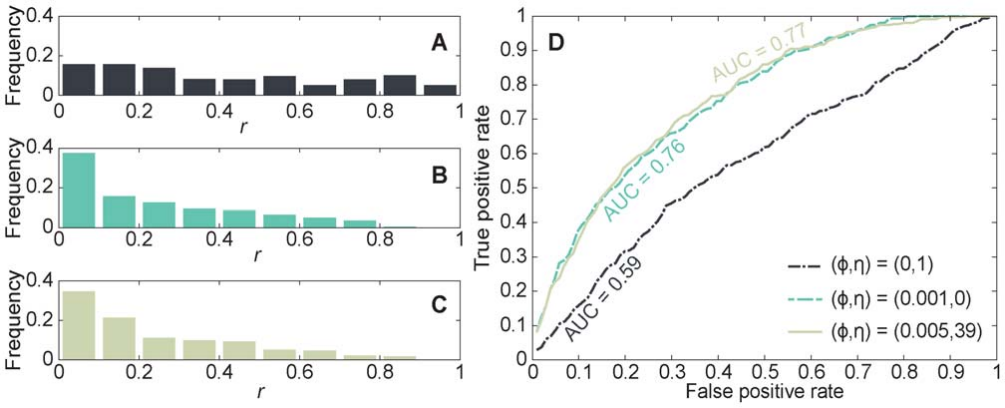
Parameter dependence of prediction performance. (A) The distributions of *r*-ratios for the known OMIM disease genes of the 40 diseases under study, at (*φ*,*η*)  =  (0,1), i.e., only gene expression levels were used to predict disease genes. (B) The distributions of *r*-ratios for the known OMIM disease genes at (*φ*,*η*)  =  (0.001,0), i.e., only the PPI network was used in the ranking. (C) The distributions of *r*-ratios for the known OMIM disease genes at (*φ*,*η*)  =  (0.005,39), (C) ROC curves for (*φ*,*η*)  =  (0.005,39), (0.001,0), and (0,1), respectively.

For the optimum (*φ*,*η*)  =  (0.005,39), and the two extreme reference values (0.001,0) and (0,1), we find average *r*-ratios of 0.246, 0.250 and 0.418. This result suggests that the known disease genes were averagely ranked top 24.6% of the candidates by integrating gene expression levels with protein interactions, whereas they ranked top 41.8% and 25%, on average, respectively, if only gene expression data or protein interaction network were utilized. In Figure 2, we show the distributions of *r*-ratios for the known OMIM disease genes of the 40 diseases and the ROC curves of our algorithm, when (*φ*,*η*) was taken as the three different values respectively. It can be seen that the ranks of gene expression levels for the disease genes are distributed almost like the average (Figure 2A), while our *s* gives rise to the enrichment of the disease genes on the top of rankings (Figure 2C). Figure 2D shows that the ROC curve for the case of *φ * =  0 is almost a diagonal line and the area under the ROC-curve (AUROC) is 0.593. When protein interactions are included in the prediction algorithm, the ROC becomes a convex curve above the diagonal line and the AUC significantly increases to 0.767. If only use PPI network (*η * =  0), the AUC is 0.764. These results indicate the significance of our approach.

**Figure 3:**
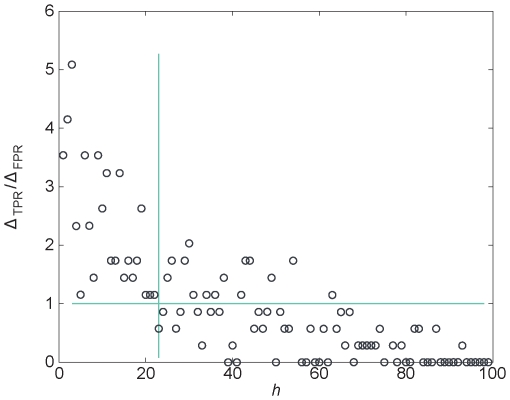
Finding a trade-off between sensitivity and specificity. The variation trend of 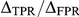
in response to changes of *h*—the number of disease genes predicted. TPR: true positive rates; FPR: false positive rates.

From the *s*-ranks of genes in each candidate set, we can predict the top *h* ones associated with the disease. In Table 1, we listed different prediction results for the known OMIM disease genes with different *h*-values. A total of 28 known disease genes were ranked first, taking a percentage of 8.1%. True positive rates (TPR) and false positive rates (FPR) suggest the sensitivity (TPR) and specificity (one minus the FPR) of our algorithm, respectively. It can be seen that with the increase of *h*, both TPR and FPR increase. That is, the increase of sensitivity is at the cost of the decrease of specificity. To find a reasonable *h* that corresponds to a good tradeoff between the sensitivity and specificity, in Figure 3, we plotted the trend of the rate at which TPR changes with respect to the change in FPR, in response to changes of *h*, *i.e.*, 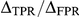
as a function of *h.* As shown, *h* = 24 appeared as a critical point where 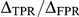 exhibits a sudden drop from values significantly larger than one to smaller than one. Since a 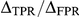-value smaller than one suggests that the gain of sensitivity is not likely to compensate the loss of specificity, *h* = 24 could be chosen as an optimal cutoff, in which the sensitivity and specificity are 60% and 76.4% respectively. In practice, there is no universal criterion for “best cutoff” but depends on the background. In our case, we think *h* = 30 is also an acceptable choice, with the sensitivity and specificity 67% and 70.4% respectively.

**Table 1:** Prediction results of our algorithm (*φ*,*η*)  =  (0.005,39) for the known OMIM disease genes of the 40 diseases under study.

h	TP	TPR	FPR	TPR/FPR
1	28	0.081	0.009	9
10	120	0.345	0.098	3.520
15	163	0.468	0.147	3.184
24	208	0.600	0.236	2.542
30	233	0.670	0.296	2.264

*h*: number of genes on the top of the candidate ranking that was predicted as disease-associated; TP: true positive numbers, i.e., number of known disease genes that was predicted as disease-associated; TPR: true positive rates; FPR: false positive rates.

Out of the 40 diseases we also study three monogenic diseases, caused by a single gene mutation. This is, of course, to evaluate method rather than to disprove that the disease is monogenetic. The other diseases are complex diseases believed to be associated to variations or dysfunctions of multiple genes. As shown in Table 2, the causing single genes of the 3 monogenic diseases, progeria, Duchenne muscular dystrophy, and cystic fibrosis, were successfully identified by our algorithm. While checking the complex diseases, we found that many disease genes with highest rankings have been reported as associated with the diseases in other sources than OMIM. For example, genes APOE, APP, PSEN1 and PSEN2 have been revealed being linked to autosomal dominant or familial early onset Alzheimer's disease by genetic studies [19]. Genome-wide association (GWA) studies have identified some top candidate genes that consistently replicate in Crohn's disease, which include NOD2 and IL23R [20]. Insulin resistance has been known strongly associated with type II diabetes, thus genes IRS1 and IRS2, which play central roles in insulin signal transmission, are important candidate genes associated with type II diabetes [21]. See Table S2 for detailed prediction results of the known disease genes.

**Table 2:** Selected prediction results for disease genes in three monogenic diseases and complex diseases, respectively.

Disease MeSH	Gene name	Gene loci	*s*-rank
Progeria	LMNA	1q21.2	4
Muscular Dystrophy, Duchenne	DMD	Xp21.2	2
Cystic Fibrosis	CFTR	7q31.2	8
Alzheimer Disease	APOE	19q13.2	3
	APP	21q21	4
	PSEN1	14q24.3	4
	PSEN2	1q31-q42	15
Crohn Disease	IL6	7p21	1
	IL23R	1p31.3	3
	NOD2	16q12	4
Diabetes Mellitus, Type 2	IL6	7p21	1
	PPARG	3p25	1
	IRS1	2q36	2
	IRS2	13q34	3

*s*-rank: ranks of candidate genes according to their *s*-values when (*φ*,*η*)  =  (0.005,39).

### Disease gene prediction when disease genes are partially known

In the last section, we assume that no genes on disease loci have been associated with the disease. Thus we only used gene expression level to represent the activity level of gene in the disease. In fact, genetic studies have uncovered plenty of links between diseases and particular chromosomal regions, while some of these disease loci have identified causative genes but the others have not yet. For example, APOE, APP, PSEN1 and PSEN2 are known Alzheimer's disease associated genes located at loci 19q13.2, 21q21, 14q24.3 and 1q31–q42, respectively. Other chromosomal regions such as 12p11.23–q13.12 and 10q24 have been identified as related with this disease, but no specific genes have got confirmed yet, hence disease genes on these loci are labeled as AD5 and AD6 respectively in the OMIM morbid map (OMIM ID 602096, 605526). Here we tried to investigate whether the known disease genes could facilitate the prediction of the unknowns.

**Figure 4:**
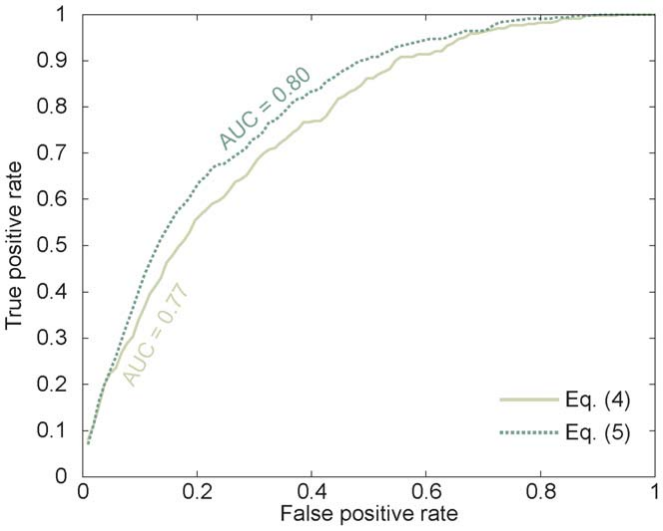
ROC curves for the predictions of disease genes. Here we restrict the analysis to diseases with at least two known associated genes.

For diseases with multiple known associated genes, we utilized partially known disease genes to predict the others. Specifically, we successively took out one gene and used the rest of the genes as input to predict this one. We modified equation (4) as follows:
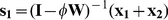
(5)where **x_1_** is the normalized vector of **d** + *η* **x** in equation (4), and vector **x_2_** was constructed such that the components corresponding to the input known genes were assigned as 1 and the other components were assigned as 0. As we did in the last section, taking (*φ*,*η*) as (0.005,39), we computed the *s*_1_-scores of genes and then ranked the candidate genes accordingly. We found that, compared with the results of last section which only used gene expression levels as input, the ranks of most disease genes went up and the *r*-ratio decreased to 21.11 (See Table S2). In Figure 4 we show the performance comparison of the predictions in situations of inputting partial known disease genes or not. It can be seen that when partial known disease genes were utilized in the prediction, the area under the ROC-curve (AUROC) increased to 0.80. These results suggest that our algorithm performed better when more information about the disease was known.

As listed in Table S3, among the diseases we studied, 123 genes have not been identified on known disease-associated chromosomal regions. Similar as we did above, we tried to predict the unknown genes using all the known disease genes. Here we present our results on Alzheimer's disease (AD) as an example. The OMIM morbid map collected 15 known AD-associated genes (see Table S2) and 12 unknown genes denoted as AD5∼AD16. Using all the known genes as input, we ranked the candidates on each chromosmal region of unknown genes by their s_1_-scores. Then starting from the top of each candidate rank, we picked a gene and conducted literature search in PubMed to explore possible links of this gene to Alzheimer's disease. We listed our predictions of AD-associated genes that have found literature supports in Table 3 and the evidences in literature as follows:

- VDR: Vitamin D3 is known to be involved in neuroprotection. Vitamin D receptor (VDR) gene can influence the affinity of vitamin D3 to its receptor and thus associated with AD [22].
- BTRC: BTRC mediates the ubiquitination and subsequent proteasomal degradation of target proteins. Defects in ubiquitin-dependent proteolysis have been shown to result in a variety of neurodegenerative diseases [23].
- TRDMT1: TRDMT1is a DNA methylation protein involved in aging-related process [24].
- PCNA: Expression of PCNA was observed in glial cells and neurons, with a trend to increased expression in association with higher burdens of Alzheimer-type pathology [25].
- ICAM1: Monocytic cell adhesion molecules are decreased in AD patients [24]. Elevated cerebrospinal fluid soluble ICAM-1 is associated with lower perfusion levels in the parietal cortex of cognitively intact elderly [27].
- NOS3: Expression of the NOS3 gene has been demonstrated in degenerating neurons and glial cells in brains with AD [28].
- CDKN2A: CDKN2A is a promising new candidate gene potentially contributing to AD susceptibility on chromosome 9p [29].
- FGFR1: Gene expression of FGFR1 was up-regulated in amyloid beta protein-injected mouse model for Alzheimer disease [30].
- S100A4: S100-mediated signal transduction pathways play an important role in nervous system function or disease, and S100A4 has been shown implicated in neurological diseases [31].
- PRDX6: Oxidative stress conditions exist in AD and peroxiredoxin 6 is an important antioxidant enzyme in human brain defenses [32].
- TF: Epistatic interaction between rs1049296 (P589S) in the transferrin gene (TF) and rs1800562 (C282Y) in the hemochromatosis gene (HFE) results in significant association with risk for AD [33].
- COX7B: Amyloidbetapeptide (A beta) is implicated in neuronal cell death in Alzheimer's disease. Studies on AD suggest that COX7B mRNA is increased in AD brains and its overexpression in cells enhances A beta(1-40)-toxicity [34].

**Table 3:** Alzheimer's disease (AD) associated genes predicted by our algorithm that have found literature supports.

	Unknown AD genes in OMIM morbid			Predicted AD-associated genes by our algorithm			
No	Gene Symbol in OMIM morbid	OMIM ID	Gene loci	Gene ID	Gene Symbol	Gene loci	*s*_1_-rank
1	AD5	602096	12p11.23–q13.12	7421	VDR	12q13.11c	3
2	AD6	605526	10q24	8945	BTRC	10q24.32a	4
3	AD7	606187	10p13	1787	TRDMT1	10p13a	9
4	AD8	607116	20p	5111	PCNA	20p12.3c	2
5	AD9	608907	19p13.2	3383	ICAM1	19p13.2c	1
6	AD10	609636	7q36	4846	NOS3	7q36.1c–q36.1d	1
7	AD11	609790	9p22.1	1029	CDKN2A	9p21.3c	2
8	AD12	611073	8p12–q22	2260	FGFR1	8p12a	1
9	AD13	611152	1q21	6275	S100A4	1q21.3c	3
10	AD14	611154	1q25	9588	PRDX6	1q25.1a	1
11	AD15	611155	3q22–q24	7018	TF	3q22.1e	1
12	AD16	300756	Xq21.3	1349	COX7B	Xq21.1a	3

Table 3 shows that almost half of the predicted disease genes are ranked first in the list of candidate genes, suggesting a good performance of our algorithm.

### Analysis of the globally top ranked genes

For each disease under study, we computed *s*_1_ for all vertices by equation (5) using gene expression levels and all known disease genes as input. Then we neglected, for the moment, the expression data and ranked genes in the protein interaction network according to their *s*_1_-values. It was found that the top genes overlapped in most diseases. For example, gene AKT1 and TP53 were ranked top 10 in 87.8% and 82.9% diseases under study, respectively.

We took out the top 200 *s*_1_-ranked genes of each disease and got 1330 genes in total, 107 of which overlapped in at least 90% diseases under study (see Table S4 for detail). (In the table we called them top ranked genes.) However, only 23 of them are disease genes of these 40 diseases. To explore the implications of the top ranked genes to diseases, we conducted gene ontology (GO) and pathway enrichment analysis. We used the P-value to quantitatively measure whether this top ranked gene group is more enriched with genes of a specific Gene ontology (GO) term or genes involved in a particular pathway than what would be expected by chance. Given significance level *α* = 0.05, a P-value smaller than *α* demonstrates low probability that the genes of same GO term or pathway appear in the group by chance. As listed in Table 4, this top ranked group is significantly enriched with genes whose GO terms are response to stimulus and stress, regulation of cell differentiation, proliferation and death, and immune process. These biological processes are highly associated with the progress of diseases, especially cancers. When mapping these genes onto KEGG pathways, we found that a total of 42 disease pathways are significantly enriched with genes in this group, 17 of which are among the 40 diseases under study (see Table S5). In addition, these top 1% *s*-ranked genes are significantly involved in 32 pathways of cellular processes, organismal systems and environmental information processing (see Table S6). It has been known that most of these pathways are related with diseases.

**Table 4:** Selected significantly enriched GO terms for the top s1-ranked genes.

GO ID	GO Term	Mapped genes	Total genes
GO:0050896	response to stimulus	68	6192
GO:0006950	response to stress	53	2538
GO:0002376	immune system process	44	1436
GO:0030154	cell differentiation	43	2008
GO:0042127	regulation of cell proliferation	40	946
GO:0010941	regulation of cell death	44	1042

All reported genes are significant with a P-value less than 0.001.

**Figure 5:**
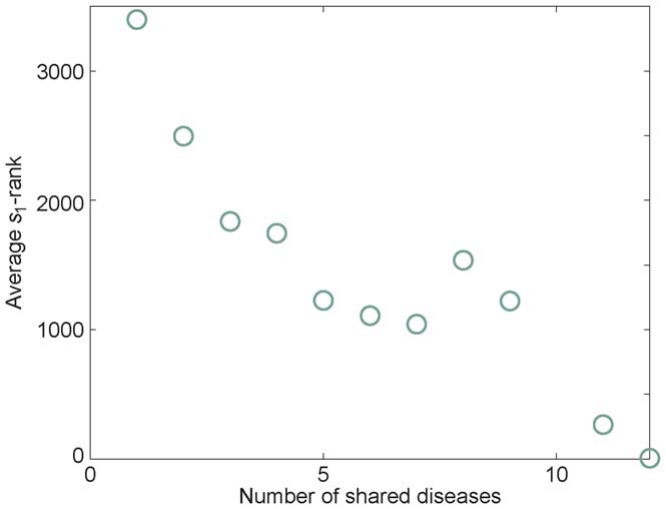
Correlation between the importance and pleiotropy. We measure the *s*_1_-score averaged over bins of the number of shared diseases for that particular gene (as a measure of the strength of pleiotropy).

Next, we studied the correlation between *s*_1_-rank and the pleiotropic effects of disease genes. Disease gene pleiotropy refers to the ability of different mutations within the same gene to cause different pathological effects. For each of the 318 known disease genes of the 40 diseases under study, we searched the OMIM morbid map and got the number of different diseases shared with this gene. Figure 5 displays the negative correlation between average *s*_1_-rank of known disease genes and the number of shared diseases (Pearson's correlation coefficient is –0.906), suggesting that our algorithm ranks genes with more pleiotropy higher. This phenomenon confirmed our observation above that the globally top ranked genes tend to be involved in multiple diseases.

To investigate whether the top ranked genes are intrinsic for diseases, for each of the 81 disease-control sets, we generated ten random counterparts of gene expression levels and known disease gene sets, respectively. Replacing vectors **x**_1_ and **x**_2_ in equation (5) with those corresponding to their random counterparts, we computed the *s*_1_-scores of genes by equation (5). As we did above, we took out the top 200 *s*_1_-ranked genes of each random counterpart. Almost all the genes appeared at least once in a top 200 list, in which only two genes overlapped in at least 35% random counterparts. In contrary to what computed from real gene expression levels and known disease gene sets of diseases, these top ranked genes exhibited very low extent of overlapping. This result suggests that only real data reflecting the activity levels of genes in disease status could help to correctly pick out the genes with the features we observed above. Thus the globally top s_1_-ranked genes are inherently correlated with diseases.

In summary, although only a small fraction (21.5%) are disease genes in the OMIM database, these globally top ranked genes are significantly involved in multiple disease processes. This is in line with previous findings that comorbidity between different diseases is linked by phenotypic interdependency (via protein interactions) and common pathophysiology (being differentially expressed in microarray data). Our result suggests that these top ranked genes could be bridges to relate different diseases with each other.

### Conclusions

This work has discussed a method to integrate microarray-based global gene expression data and genome scale protein-protein interaction network for the prioritization of candidate disease genes. According to the observation that disease genes tend to be close to other disease genes in the associated protein network, we proposed a score inspired by the Katz centrality. This score needs to be calibrated by only two parameters. These parameters have a clear biological interpretation so their optimal values can give us some further insights. The first parameter *φ* sets the relative importance of the difference in expression level and closeness in the protein interaction network. The second parameter *η* represents chance for a node that is not differentially expressed to be a disease gene. The optimum is reached for (*φ*,*η*)  =  (0.005,39), which is well in the interior of the parameter space in both dimensions—0≤ *φ* <0.01 and 0≤ *η* <0.01. This means that both the protein interaction network and the differential expression contain information that can be exploited in disease-gene ranking, as hypothesized. On the other hand, we see that putting *Ø* = 0 worsen the performance much more than putting *η* = 0, which suggest that there is more information for the benefit of predicting unknown disease genes in the interaction compared with the microarray data, at least with our setup. Furthermore, we were able to increase our method's performance by including partial information about known disease genes. Also, when we did not consider specific gene loci and ranked all genes globally by our score, we could identify genes that show high extent of pleiotropy and participate in the physiological pathogenic processes of many diseases [35]. In addition, the successful identification of the common genes involved in many diseases in the context of network indicates the phenotypic interdependency, cooccurrence and shared pathophysiology between different disorders. This study provides a novel, effective and easy-implemented algorithm for the prioritization of candidate disease genes. It can also be used to compare the connection between pathological phenotypes through their common genetic factors.

## Materials and Methods

### Gene expression data collection and integration

We collected human microarray datasets from the NCBI Gene Expression Omnibus (GEO) [36] and restricted to using only those curated and reported in the GEO Datasets (GDS). Our criteria for the selection of a dataset are as follows:

1. It used one of the four most common platforms: HG_U95A, HG_U95Av2, HG_U133A, and HG_U133_Plus2.
2. It was assigned to human disease conditions, with healthy samples as the control condition. In addition, at least one disease gene of this disease is known from the OMIM database. The samples were not treated by drugs.
3. It did not include time-series data.
4. It included at least four disease samples and four control samples.

A total of 58 datasets was found to satisfy the criteria. We combined the samples of one disease and the normal samples in the same experiment into a disease-control set. Since GEO contains some experiments that include gene expression measurements for more than one disease, 81 disease-control sets were obtained from the 58 datasets. Mapping the disease description in the GDS curation to their MeSH (Medical Subject Headings) terms corresponded to 40 distinct diseases (see Table S1).

To integrate gene expression data from different platforms, we mapped the probe sets of the platforms to Entrez Gene ID. This process yielded a set of 9308 genes common to all four platforms for our further study. For each gene in a dataset, we calculated the average expression level for probe sets associated to this gene, and converted the expression value to its rank among expression values of the sample. The rank transformation allows for the direct comparison of gene expression levels across various microarray experiments [37], [38]. To identify differentially expressed genes, for each gene in a disease-control set, we calculated the log ratio of the average rank of disease samples versus the average rank of control samples. We take the absolute value of the log ratio as a measure of the activity level of the gene in this disease.

### Protein-protein interaction data

Protein interactions between human proteins were downloaded from the version 8.3 of STRING [39]. STRING includes both physical and functional interactions integrated from numerous sources, including experimental repositories, computational prediction methods and public text collections. It uses a scoring system to weight the evidence of each interaction. STRING includes the interactions between 14532 proteins of human genome. We normalized the interaction scores in STRING to the interval [0,1].

### Disease-gene dataset

We searched the Morbid map of the Online Mendelian Inheritance in Man (OMIM) database [40] and identified 359 genes associated with the 40 distinct diseases in our microarray data, in which one disease was associated with at least one gene. As listed in Table S2, the disease with the most known causing genes is cardiomyopathy, with 32 disease genes known. A total of 318 of the 359 genes were found to present in the protein-protein interaction network constructed from STRING, and these genes were used to validate our algorithm (see Table S2).

### Candidate genes

We downloaded human gene location data from the FTP server of NCBI's MapViewer [41]. This source includes the chromosomal locations and chromosomal base pair ranges of human genes. For each of the 318 known disease genes, we determined a set of about 100 candidate genes, including this disease gene, which locate at, or near to the cytogentic loci of the disease gene.

### Disease gene prediction

Most of our algorithm is already detailed in the section Results and Discussion. We mention, however, that we solved equation (4) by Jacobi iteration algorithm. Furthermore, for each disease-control dataset *k*, an *s*-vector was calculated by equation (4). In cases when one disease corresponds to more than one experiment (disease-control datasets), the score vectors for a given disease were added together to obtain a combined *s*-score. Then the genes in each candidate gene set of a disease can be ranked according to their *s*-cores, while the top *h* genes in the ranking could be predicted as associated with this disease.

### Performance measure

The known disease genes in the OMIM database were used to determine parameters *φ* and *η*, as well as to assess the performance of our algorithm. For a known disease gene in a candidate gene set of size *N*, if its *s*-rank calculated by our algorithm is *r*, then its *r*-ratio defined as *r/N*, could reflect how strong this gene is predicted as a disease gene. We determined parameters *φ* and *η* as those minimized the average *r*-ratios of the known OMIM disease genes.

We then applied the receiver operating characteristics (ROC) analysis [42] to evaluate our algorithm. We took the top *h* genes in each of our candidate gene rankings as disease genes (positive). Changing *h* from 1 to 100, we computed the true positive rates (TPR) and false positive rates (FPR) of our predictions. Then a ROC curve is obtained by plotting TPR versus FPR for the *h*-values. A ROC curve gives an overview of the overall performance of a classifier. When comparing ROC-curves of different classifiers, good curves lie closer to the top left corner and the worst case is a diagonal line that represents a strategy of random guessing. The total area under the ROC-curve (AUROC) is a measure of the performance of the classifier. The area lies in the interval [0.5,1] and larger area indicates better performance. On the other hand, the values of TPR and FPR suggest the sensitivity and specificity of the classifier, respectively. Larger TPR and smaller FPR correspond to both higher sensitivity and specificity. Usually, the increase of sensitivity is at the cost of the decrease of specificity. In our case, with the increase of *h*, both TPR and FPR increase. Only when the increase of TPR is faster than that of FPR, i.e. 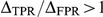, taking the larger *h* is cost-efficient. Thus the optimal trade-off value of *h* satisfies:
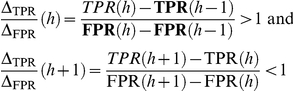
is a natural cutoff position in the candidate ranking that corresponds to a optimum tradeoff between the sensitivity and specificity.

### Pathway data and pathway enrichment analysis

We downloaded pathway data from the FTP service of KEGG [43] (Kyoto Encyclopedia of Genes and Genomes) on June 21, 2011. The KEGG PATHWAY section is a collection of manually drawn pathway maps representing the information on the molecular interaction and reaction networks. The “hsa_pathway.list” file in this section includes a list of the known proteins in *H. sapiens*' genome and the corresponding pathways that they are involved in.

We used pathway enrichment analysis [44] to determine whether a pathway is significantly enriched with a group of genes. Specifically, we compare with a hypergeometric cumulative distribution [45] to measure whether a pathway is more enriched with the gene group under study than would be expected by chance. Given significance level *α* = 0.05, a P-value smaller than *α* suggests a low probability that the gene group appear in the pathway by chance, *i.e.*, the pathway can be regarded as being significantly influenced by this group of genes under the null-hypothesis of a hypergeometric cumulative distribution.

### Generating random counterparts of gene expression levels of diseases and known disease gene sets

For each disease-control set, we selected a pair of genes randomly and exchanged their activity values in the disease (the log ratio of the average rank of disease samples versus the average rank of control samples). Repeating this process a sufficiently large number of times gave us a randomly reshuffled vector of gene expression levels for the disease-control set, which we used as a random reference of gene expression levels for this disease.

As the known disease genes of the 40 diseases under study are at least 1 and at most 32, we generated an array of random integers chosen from the continuous uniform distribution over the interval [1], [32] to simulate the numbers of disease genes. Then, for each random number *R* in the array, we selected *R* genes randomly in the protein interaction network as random counterparts of known causing genes of the disease.

## Supporting Information

Table S1 Description of miroarray datasets under study.
(DOCX)

Table S2 Known OMIM genes associated with the 40 diseases under study and their ranks in the candidate gene sets. e–rank: ranks of candidate genes according to the absolute values of log ratio for the expression levels [equation 3, (φ,η)  =  (0,1)]; p–rank: ranks of candidate genes according to protein interactions [equation 3, (φ,η)  =  (0.001,0)]; s–rank: ranks of candidate genes according to their *s*-scores [equation 3, (φ,η)  =  (0.005, 39)], when gene expression levels were used as input activity level of genes in the disease; s1–rank: ranks of candidate genes according to their *s*_1_-scores [equation 4, (φ,η)  =  (0.005, 39)], when gene expression levels and the other known causative genes of the disease were used as input activity level of genes in the disease.
(DOCX)

Table S3 Unidentified genes on known chromosomal regions associated with the diseases under study, from OMIM morbid map.
(DOCX)

Table S4 List of the top ranked genes, i.e., the top 200 *s*_1_-ranked genes in over 90% diseases under study.
(DOCX)

Table S5 Disease pathways significantly enriched with the top ranked genes.
(DOCX)

Table S6 Pathways significantly enriched with the top ranked genes.
(DOCX)
