# Supplementary material for: Ranking Candidate Disease Genes from Gene Expression and Protein Interaction: A Katz-Centrality Based Approach
Source: PLoS One. 2011 Sep 2;6(9):e24306. doi: 10.1371/journal.pone.0024306 (PMC3166320; doi:10.1371/journal.pone.0024306)
Supplement: Table S1 — Description of miroarray datasets under study. (DOCX) [file pone.0024306.s001.docx]

| Disease–  control set | Dataset | Platform | Disease description in dataset | Disease MeSH term | Positive samples | Control samples |
| --- | --- | --- | --- | --- | --- | --- |
| 1 | GDS2142 | HG_U133A | Cystic fibrosis,mild | Cystic Fibrosis | 4 | 10 |
| 2 | GDS2142 | HG_U133A | Cystic fibrosis,severe | Cystic Fibrosis | 5 | 10 |
| 3 | GDS3027 | HG_U133A | Duchenne muscular dystrophy | Muscular Dystrophy, Duchenne | 23 | 14 |
| 4 | GDS563 | HG_U95Av2 | Duchenne muscular dystrophy | Muscular Dystrophy, Duchenne | 12 | 11 |
| 5 | GDS1956 | HG_U133A | muscle disease 7:Becker muscular dystrophy | Muscular Dystrophy, Duchenne | 5 | 18 |
| 6 | GDS1956 | HG_U133A | muscle disease 8:Duchenne muscular dystrophy | Muscular Dystrophy, Duchenne | 10 | 18 |
| 7 | GDS1503 | HG_U133A | Hutchinson–Gilford progeria syndrome | Progeria | 9 | 9 |
| 8 | GDS1726 | HG_U95Av2 | HIV encephalitis | AIDS Dementia Complex | 16 | 12 |
| 9 | GDS2795 | HG_U133_Plus_2 | Alzheimer's disease | Alzheimer Disease | 10 | 10 |
| 10 | GDS810 | HG_U133A | Alzheimer's disease 1,incipient | Alzheimer Disease | 7 | 9 |
| 11 | GDS810 | HG_U133A | Alzheimer's disease 2,moderate | Alzheimer Disease | 8 | 9 |
| 12 | GDS810 | HG_U133A | Alzheimer's disease 3,severe | Alzheimer Disease | 7 | 9 |
| 13 | GDS1956 | HG_U133A | muscle disease 3:amyotophic lateral sclerosis | Amyotrophic Lateral Sclerosis | 9 | 18 |
| 14 | GDS711 | HG_U95Av2 | pauciarticular rheumatoid arthritis(RA1) | Arthritis, Juvenile Rheumatoid | 10 | 11 |
| 15 | GDS711 | HG_U95Av2 | polyarticular rheumatoid arthritis(RA2) | Arthritis, Juvenile Rheumatoid | 25 | 11 |
| 16 | GDS2126 | HG_U95A | Rheumatoid arthritis | Arthritis, Rheumatoid | 5 | 5 |
| 17 | GDS266 | HG_U133A | Asthma | Asthma | 20 | 9 |
| 18 | GDS3711 | HG_U133A | Asthma | Asthma | 9 | 7 |
| 19 | GDS261 | HG_U95Av2 | asthma | Asthma | 5 | 6 |
| 20 | GDS2880 | HG_U133A | Clear cell renal cell carcinoma | Carcinoma, Renal Cell | 10 | 10 |
| 21 | GDS505 | HG_U133A | Renal clear cell carcinoma | Carcinoma, Renal Cell | 9 | 8 |
| 22 | GDS2520 | HG_U95Av2 | head and neck squamous carcinoma | Carcinoma, Squamous Cell | 22 | 22 |
| 23 | GDS1062 | HG_U133A | metastasis of oral Squamous cell carcinoma | Carcinoma, Squamous Cell | 19 | 8 |
| 24 | GDS2200 | HG_U133A | squamous cell carcinoma | Carcinoma, Squamous Cell | 5 | 6 |
| 25 | GDS2154 | HG_U133_Plus_2 | dilated cardiomyopathy | Cardiomyopathy | 8 | 4 |
| 26 | GDS651 | HG_U133_Plus_2 | idiopathic dilated cardiomyopathy | Cardiomyopathy | 15 | 11 |
| 27 | GDS651 | HG_U133_Plus_2 | Ischemic cardiomyopathy | Cardiomyopathy | 11 | 11 |
| 28 | GDS1362 | HG_U133A | Ischemic cardiomyopathy | Cardiomyopathy | 10 | 6 |
| 29 | GDS1362 | HG_U133A | nonischemic cardiomyopathy | Cardiomyopathy | 21 | 6 |
| 30 | GDS2947 | HG_U133_Plus_2 | Colorectal adenoma | Colorectal Neoplasms | 32 | 32 |
| 31 | GDS2609 | HG_U133_Plus_2 | colorectal cancer,early onset | Colorectal Neoplasms | 12 | 10 |
| 32 | GDS2642 | HG_U95Av2 | Crohn's disease | Crohn Disease | 19 | 4 |
| 33 | GDS1615 | HG_U133A | Crohn's disease | Crohn Disease | 59 | 42 |
| 34 | GDS3345 | HG_U95Av2 | depression | Depression | 11 | 15 |
| 35 | GDS1956 | HG_U133A | muscle disease 2:juvenile dermatomyositis | Dermatomyositis | 21 | 18 |
| 36 | GDS3347 | HG_U95Av2 | Type 2 diabetes | Diabetes Mellitus, Type 2 | 10 | 10 |
| 37 | GDS3681 | HG_U95Av2 | Type 2 diabetes | Diabetes Mellitus, Type 2 | 10 | 10 |
| 38 | GDS1321 | HG_U133A | esophageal adenocarcinoma | Esophageal Neoplasms | 8 | 8 |
| 39 | GDS1112 | HG_U95Av2 | Glaucoma | Glaucoma | 7 | 6 |
| 40 | GDS1962 | HG_U133_Plus_2 | Glioma grade II | Glioma | 45 | 23 |
| 41 | GDS1962 | HG_U133_Plus_2 | Glioma grade III | Glioma | 31 | 23 |
| 42 | GDS1962 | HG_U133_Plus_2 | Glioma grade IV | Glioma | 81 | 23 |
| 43 | GDS2887 | HG_U133_Plus_2 | Huntington's disease | Huntington Disease | 10 | 10 |
| 44 | GDS1331 | HG_U133A | Huntington's disease | Huntington Disease | 12 | 14 |
| 45 | GDS946 | HG_U133A | familial combined hyperlipidemia (FCHL) | Hyperlipidemia, Familial Combined | 12 | 12 |
| 46 | GDS2200 | HG_U133A | actinic keratosis | Keratosis, Actinic | 4 | 6 |
| 47 | GDS3057 | HG_U133A | Acute myeloid leukemia | Leukemia, Myeloid, Acute | 26 | 38 |
| 48 | GDS3257 | HG_U133A | lung adenocarcinoma | Lung Neoplasms | 58 | 49 |
| 49 | GDS1650 | HG_U95Av2 | Pulmonary adenocarcinoma | Lung Neoplasms | 20 | 19 |
| 50 | GDS1312 | HG_U133A | Squamous lung cancer | Lung Neoplasms | 5 | 5 |
| 51 | GDS1971 | HG_U133A | complicated malaria | Malaria | 5 | 5 |
| 52 | GDS1971 | HG_U133A | Uncomplicated malaria | Malaria | 5 | 5 |
| 53 | GDS1375 | HG_U133A | primary malignant melanoma | Melanoma | 45 | 7 |
| 54 | GDS1220 | HG_U133A | malignant pleural mesothelioma (MPM) tumors | Mesothelioma | 44 | 10 |
| 55 | GDS1392 | HG_U133A | Myelodysplastic syndrome | Myelodysplastic Syndromes | 19 | 9 |
| 56 | GDS2118 | HG_U133_Plus_2 | Myelodysplastic syndromes | Myelodysplastic Syndromes | 55 | 11 |
| 57 | GDS1480 | HG_U133A | Obesity | Obesity | 14 | 14 |
| 58 | GDS268 | HG_U133A | Obesity | Obesity | 8 | 8 |
| 59 | GDS268 | HG_U133A | Obesity | Obesity | 8 | 8 |
| 60 | GDS3601 | HG_U95A | Obesity | Obesity | 9 | 10 |
| 61 | GDS3602 | HG_U95Av2 | Obesity | Obesity | 10 | 10 |
| 62 | GDS2126 | HG_U95A | osteoarthritis | Osteoarthritis | 5 | 5 |
| 63 | GDS1956 | HG_U133A | muscle disease 4:spastic paraplegia | Paraplegia | 4 | 18 |
| 64 | GDS2519 | HG_U133A | Parkinson’s disease | Parkinson Disease | 50 | 22 |
| 65 | GDS2519 | HG_U133A | Parkinson’s disease | Parkinson Disease | 50 | 33 |
| 66 | GDS3104 | HG_U133_Plus_2 | Insulin–resistant polycystic ovary syndrome | Polycystic Ovary Syndrome | 16 | 13 |
| 67 | GDS2084 | HG_U133A | Polycystic ovary syndrome | Polycystic Ovary Syndrome | 8 | 7 |
| 68 | GDS2545 | HG_U95Av2 | metastatic prostate tumor | Prostatic Neoplasms | 25 | 18 |
| 69 | GDS2545 | HG_U95Av2 | metastatic prostate tumor | Prostatic Neoplasms | 25 | 63 |
| 70 | GDS2545 | HG_U95Av2 | primary prostate tumor | Prostatic Neoplasms | 65 | 18 |
| 71 | GDS2545 | HG_U95Av2 | primary prostate tumor | Prostatic Neoplasms | 65 | 63 |
| 72 | GDS1746 | HG_U133A | prostate tumors | Prostatic Neoplasms | 22 | 8 |
| 73 | GDS1209 | HG_U133A | Sarcoma | Sarcoma | 39 | 15 |
| 74 | GDS1917 | HG_U133_Plus_2 | schizophrenia | Schizophrenia | 14 | 14 |
| 75 | GDS3345 | HG_U95Av2 | schizophrenia | Schizophrenia | 13 | 15 |
| 76 | GDS1376 | HG_U133A | Essential thrombocythemia | Thrombocythemia, Essential | 6 | 8 |
| 77 | GDS1732 | HG_U133_Plus_2 | Papillary thyroid cancer | Thyroid Neoplasms | 7 | 7 |
| 78 | GDS1665 | HG_U133_Plus_2 | Papillary thyroid carcinoma | Thyroid Neoplasms | 9 | 9 |
| 79 | GDS1479 | HG_U133A | bladder carcinoma––superficial transitional cell carcinomas with muscle invasive carcinomas (mTCC)(P3) | Urinary Bladder Neoplasms | 13 | 9 |
| 80 | GDS1479 | HG_U133A | bladder carcinoma––superficial transitional cell carcinomas with surrounding carcinoma in situ (CIS) lesions(P1) | Urinary Bladder Neoplasms | 13 | 9 |
| 81 | GDS1479 | HG_U133A | bladder carcinoma––superficial transitional cell carcinomas without surrounding carcinoma in situ (CIS) lesions(P2) | Urinary Bladder Neoplasms | 15 | 9 |
