# Supplementary material for: Ranking Candidate Disease Genes from Gene Expression and Protein Interaction: A Katz-Centrality Based Approach
Source: PLoS One. 2011 Sep 2;6(9):e24306. doi: 10.1371/journal.pone.0024306 (PMC3166320; doi:10.1371/journal.pone.0024306)
Supplement: Table S2 — Known OMIM genes associated with the 40 diseases under study and their ranks in the candidate gene sets. e–rank: ranks of candidate genes according to the absolute values of log ratio for the expression levels [equation 3, (φ,η) = (0,1)]; p–rank: ranks of candidate genes according to protein interactions [equation 3, (φ,η) = (0.001,0)]; s–rank: ranks of candidate genes according to their s-scores [equation 3, (φ,η) = (0.005, 39)], when gene expression levels were used as input activity level of genes in the disease; s1–rank: ranks of candidate genes according to their s 1-scores [equation 4, (φ,η) = (0.005, 39)], when gene expression levels and the other known causative genes of the disease were used as input activity level of genes in the disease. (DOCX) [file pone.0024306.s002.docx]

| No | Disease MeSH | OMIM ID | Gene Name | Gene Loci | e–rank | p–rank | s–Rank | s_1_–rank |
| --- | --- | --- | --- | --- | --- | --- | --- | --- |
| 1 | Cystic Fibrosis | 602421 | CFTR | 7q31.2 | 73 | 5 | 8 | – |
| 2 | Muscular Dystrophy, Duchenne | 300377 | DMD | Xp21.2 | 4 | 4 | 2 | – |
| 3 | Progeria | 150330 | LMNA | 1q21.2 | 10 | 2 | 4 | – |
| 4 | AIDS Dementia Complex | 142840 | HLA–C | 6p21.3 | 3 | 1 | 1 | 1 |
|  |  | 601373 | CCR5 | 3p21 | 79 | 1 | 1 | 2 |
|  |  | 601267 | CCR2 | 3p21 | 76 | 3 | 4 | 2 |
|  |  | 182283 | CCL3 | 17q12 | 28 | 7 | 3 | 6 |
|  |  | 187011 | CCL5 | 17q11.2–q12 | 83 | 5 | 4 | 6 |
|  |  | 158105 | CCL2 | 17q11.2–q12 | 23 | 3 | 3 | 7 |
|  |  | 601156 | CCL11 | 17q21.1–q21.2 | 74 | 13 | 13 | 11 |
|  |  | 604672 | CD209 | 19p13.3 | 18 | 23 | 19 | 16 |
|  |  | 601395 | CCL3L1 | 17q11.2 | 13 | 33 | 21 | 20 |
| 5 | Alzheimer Disease | 163729 | NOS3 | 7q36 | 50 | 2 | 2 | 3 |
|  |  | 107741 | APOE | 19q13.2 | 4 | 3 | 3 | 3 |
|  |  | 191840 | PLAU | 10q24 | 97 | 3 | 3 | 3 |
|  |  | 104760 | APP | 21q21 | 23 | 3 | 4 | 3 |
|  |  | 235200 | BMP2 | 6p21.3 | 69 | 4 | 4 | 4 |
|  |  | 104311 | PSEN1 | 14q24.3 | 52 | 3 | 4 | 4 |
|  |  | 235200 | HFE | 6p21.3 | 87 | 2 | 5 | 5 |
|  |  | 103950 | A2M | 12p13.3–p12.3 | 53 | 8 | 10 | 9 |
|  |  | 600759 | PSEN2 | 1q31–q42 | 81 | 9 | 15 | 10 |
|  |  | 606989 | MPO | 17q23.1 | 81 | 6 | 12 | 12 |
|  |  | 106180 | ACE | 17q23 | 88 | 13 | 20 | 17 |
|  |  | 602710 | APBB2 | 4p14 | 45 | 34 | 37 | 32 |
|  |  | 602403 | BLMH | 17q11.2 | 28 | 40 | 39 | 33 |
|  |  | 602005 | SORL1 | 11q23.2–q24.2 | 19 | 63 | 43 | 39 |
|  |  | 608254 | PAXIP1 | 7q36 | 79 | 63 | 80 | 78 |
| 6 | Amyotrophic Lateral Sclerosis | 147450 | SOD1 | 21q22.1 | 1 | 1 | 1 | 1 |
|  |  | 602533 | PARK7 | 1p36 | 1 | 9 | 1 | 2 |
|  |  | 137070 | FUS | 16p11.2 | 29 | 6 | 13 | 12 |
|  |  | 601143 | DCTN1 | 2p13 | 99 | 6 | 18 | 12 |
|  |  | 605692 | TRPM7 | 15q21 | 6 | 43 | 21 | 19 |
|  |  | 605078 | TARDBP | 1p36.2 | 39 | 35 | 39 | 24 |
|  |  | 604061 | 9–Sep | 17q25 | 9 | 57 | 26 | 27 |
|  |  | 606352 | ALS2 | 2q33 | 13 | 62 | 36 | 31 |
|  |  | 170710 | PRPH | 12q12–q13 | 52 | 29 | 41 | 32 |
|  |  | 105850 | ANG | 14q11.2 | 58 | 29 | 35 | 33 |
|  |  | 608465 | SETX | 9q34 | 82 | 35 | 64 | 37 |
|  |  | 609512 | CHMP2B | 3p11.2 | 42 | 54 | 55 | 45 |
|  |  | 162230 | NEFH | 22q12.2 | 96 | 40 | 50 | 47 |
|  |  | 602432 | OPTN | 10p15–p14 | 87 | 30 | 49 | 49 |
|  |  | 609390 | FIG4 | 6q21 | 41 | 77 | 71 | 73 |
| 7 | Arthritis, Juvenile Rheumatoid | 147620 | IL6 | 7p21 | 6 | 1 | 1 | 1 |
|  |  | 153620 | MIF | 22q11.2 | 27 | 44 | 38 | 38 |
| 8 | Arthritis, Rheumatoid | 600005 | CIITA | 16p13 | 73 | 5 | 7 | 7 |
|  |  | 142857 | HLA–DRB1 | 6p21.3 | 15 | 25 | 9 | 11 |
|  |  | 600716 | PTPN22 | 1p13 | 90 | 10 | 12 | 11 |
|  |  | 605554 | CD244 | 1q22 | 19 | 16 | 17 | 16 |
|  |  | 600558 | STAT4 | 2q32.2–q32.3 | 14 | 24 | 18 | 17 |
|  |  | 605347 | PADI4 | 1p36.13 | 15 | 38 | 27 | 26 |
|  |  | 601022 | NFKBIL1 | 6p21.3 | 42 | 61 | 55 | 55 |
| 9 | Asthma | 161561 | IL12B | 5q31.1–q33.1 | 67 | 4 | 4 | 6 |
|  |  | 604895 | TBX21 | 17q21.3 | 41 | 10 | 7 | 7 |
|  |  | 109690 | ADRB2 | 5q32–q34 | 24 | 6 | 7 | 8 |
|  |  | 601156 | CCL11 | 17q21.1–q21.2 | 97 | 13 | 12 | 11 |
|  |  | 152390 | ALOX5 | 10q11.2 | 66 | 16 | 15 | 14 |
|  |  | 601525 | CHI3L1 | 1q32.1 | 11 | 23 | 15 | 15 |
|  |  | 192020 | SCGB1A1 | 11q12.3–q13.1 | 48 | 17 | 20 | 16 |
|  |  | 601690 | PLA2G7 | 6p21.2–p12 | 81 | 17 | 23 | 17 |
|  |  | 604459 | IRAK3 | 12q14.3 | 59 | 28 | 27 | 20 |
|  |  | 604687 | PTGDR | 14q22.1 | 91 | 21 | 27 | 21 |
|  |  | 176804 | PTGER2 | 14q22 | 8 | 43 | 22 | 23 |
|  |  | 607796 | PHF11 | 13q14.1 | 42 | 45 | 43 | 33 |
|  |  | 605238 | HNMT | 2q22 | 19 | 40 | 44 | 35 |
|  |  | 606531 | SCGB3A2 | 5q31–q34 | 52 | 52 | 44 | 37 |
|  |  | 608595 | NPSR1 | 7p15–p14 | 50 | 43 | 50 | 38 |
|  |  | 158375 | MUC7 | 4q13–q21 | 39 | 38 | 45 | 40 |
| 10 | Carcinoma, Renal Cell | 164860 | MET | 7q31 | 5 | 4 | 2 | 2 |
|  |  | 142410 | HNF1A | 12q24.2 | 29 | 4 | 8 | 7 |
|  |  | 189907 | HNF1B | 17q12 | 10 | 10 | 11 | 8 |
|  |  | 601982 | OGG1 | 3p26.2 | 16 | 20 | 13 | 14 |
|  |  | 608537 | VHL | 3p26–p25 | 91 | 10 | 15 | 14 |
|  |  | 314310 | TFE3 | Xp11.22 | 19 | 13 | 15 | 15 |
|  |  | 602773 | DIRC2 | 3q21 | 27 | 53 | 44 | 40 |
|  |  | 603046 | RNF139 | 8q24.1 | 40 | 49 | 45 | 40 |
|  |  | 179755 | PRCC | 1q21 | 47 | 53 | 52 | 43 |
| 11 | Carcinoma, Squamous Cell | 134637 | FAS | 10q24.1 | 74 | 2 | 2 | 2 |
|  |  | 601566 | ING1 | 13q34 | 77 | 27 | 30 | 30 |
| 12 | Cardiomyopathy | 191044 | TNNI3 | 19q13.4 | 1 | 14 | 2 | 2 |
|  |  | 102540 | ACTC1 | 15q14 | 19 | 5 | 4 | 2 |
|  |  | 150330 | LMNA | 1q21.2 | 9 | 2 | 4 | 3 |
|  |  | 300377 | DMD | Xp21.2 | 23 | 4 | 5 | 3 |
|  |  | 104311 | PSEN1 | 14q24.3 | 54 | 3 | 4 | 4 |
|  |  | 193065 | VCL | 10q22.1–q23 | 53 | 4 | 5 | 5 |
|  |  | 191045 | TNNT2 | 1q32 | 9 | 9 | 10 | 5 |
|  |  | 191010 | TPM1 | 15q22.1 | 28 | 9 | 10 | 6 |
|  |  | 188840 | TTN | 2q31 | 3 | 14 | 10 | 7 |
|  |  | 102573 | ACTN2 | 1q42–q43 | 17 | 9 | 7 | 8 |
|  |  | 160781 | MYL2 | 12q23–q24.3 | 23 | 18 | 15 | 8 |
|  |  | 605906 | LDB3 | 10q22.2–q23.3 | 3 | 32 | 9 | 9 |
|  |  | 160760 | MYH7 | 14q12 | 3 | 44 | 12 | 9 |
|  |  | 600958 | MYBPC3 | 11p11.2 | 2 | 36 | 16 | 9 |
|  |  | 600824 | CSRP3 | 11p15.1 | 3 | 34 | 13 | 10 |
|  |  | 600759 | PSEN2 | 1q31–q42 | 74 | 9 | 12 | 11 |
|  |  | 191040 | TNNC1 | 3p21.3–p14.3 | 20 | 27 | 26 | 11 |
|  |  | 172405 | PLN | 6q22.1 | 10 | 17 | 16 | 15 |
|  |  | 604488 | TCAP | 17q12 | 4 | 49 | 31 | 16 |
|  |  | 103220 | SLC25A4 | 4q35 | 15 | 17 | 17 | 17 |
|  |  | 125671 | DSG2 | 18q12.1–q12.2 | 6 | 40 | 20 | 20 |
|  |  | 602743 | PRKAG2 | 7q36 | 14 | 50 | 32 | 23 |
|  |  | 300394 | TAZ | Xq28 | 47 | 35 | 36 | 28 |
|  |  | 601411 | SGCD | 5q33 | 70 | 30 | 39 | 28 |
|  |  | 600163 | SCN5A | 3p21 | 16 | 28 | 30 | 29 |
|  |  | 607440 | FKTN | 9q31 | 58 | 40 | 53 | 32 |
|  |  | 603646 | COX15 | 10q24 | 14 | 54 | 34 | 35 |
|  |  | 606566 | MYLK2 | 20q13.3 | 14 | 48 | 39 | 36 |
|  |  | 601439 | ABCC9 | 12p12.1 | 56 | 57 | 67 | 46 |
|  |  | 613121 | NEXN | 1p32–p31 | 23 | 73 | 48 | 48 |
|  |  | 188380 | TMPO | 12q22 | 93 | 50 | 66 | 48 |
|  |  | 603550 | EYA4 | 6q23 | 69 | 71 | 88 | 66 |
| 13 | Colorectal Neoplasms | 164730 | AKT1 | 14q32.3 | 46 | 1 | 1 | 1 |
|  |  | 600040 | BAX | 19q13.3–q13.4 | 26 | 2 | 1 | 1 |
|  |  | 168461 | CCND1 | 11q13 | 17 | 1 | 1 | 1 |
|  |  | 602700 | EP300 | 22q13 | 78 | 1 | 1 | 1 |
|  |  | 171834 | PIK3CA | 3q26.3 | 60 | 1 | 1 | 1 |
|  |  | 603030 | TLR4 | 9q32–q33 | 29 | 1 | 1 | 1 |
|  |  | 191170 | TP53 | 17p13.1 | 20 | 1 | 1 | 1 |
|  |  | 134934 | FGFR3 | 4p16.3 | 28 | 1 | 2 | 2 |
|  |  | 116806 | CTNNB1 | 3p22–p21.3 | 38 | 1 | 1 | 3 |
|  |  | 164757 | BRAF | 7q34 | 84 | 6 | 10 | 4 |
|  |  | 190182 | TGFBR2 | 3p22 | 6 | 5 | 4 | 5 |
|  |  | 120436 | MLH1 | 3p21.3 | 35 | 5 | 5 | 5 |
|  |  | 185535 | TACSTD1 | 2p21 | 1 | 24 | 1 | 6 |
|  |  | 603028 | TLR2 | 4q32 | 74 | 4 | 6 | 6 |
|  |  | 602932 | SMAD7 | 18q21.1 | 86 | 5 | 7 | 6 |
|  |  | 172411 | PLA2G2A | 1p35 | 6 | 25 | 6 | 7 |
|  |  | 609309 | MSH2 | 2p22–p21 | 33 | 11 | 15 | 8 |
|  |  | 600678 | MSH6 | 2p16 | 27 | 9 | 17 | 8 |
|  |  | 602860 | BUB1B | 15q15 | 12 | 9 | 10 | 10 |
|  |  | 611731 | APC | 5q21–q22 | 56 | 10 | 11 | 10 |
|  |  | 604025 | AXIN2 | 17q24 | 26 | 12 | 14 | 13 |
|  |  | 602452 | BUB1 | 2q14 | 36 | 13 | 16 | 14 |
|  |  | 600259 | PMS2 | 7p22 | 67 | 26 | 30 | 21 |
|  |  | 607273 | FLCN | 17p11.2 | 19 | 49 | 36 | 35 |
|  |  | 604395 | MLH3 | 14q24.3 | 90 | 36 | 59 | 37 |
|  |  | 120470 | DCC | 18q21.3 | 97 | 36 | 44 | 38 |
|  |  | 604584 | PDGFRL | 8p22–p21.3 | 67 | 40 | 55 | 53 |
|  |  | 600258 | PMS1 | 2q31–q33 | 66 | 78 | 83 | 65 |
|  |  | 610290 | GALNT12 | 9q22 | 34 | 73 | 70 | 72 |
| 14 | Crohn Disease | 147620 | IL6 | 7p21 | 3 | 1 | 1 | 1 |
|  |  | 607562 | IL23R | 1p31.3 | 14 | 6 | 3 | 3 |
|  |  | 605956 | NOD2 | 16q12 | 14 | 10 | 4 | 3 |
| 15 | Depression | 182135 | HTR2A | 13q14–q21 | 50 | 21 | 31 | – |
| 16 | Dermatomyositis | 120940 | C9 | 5p13 | 93 | 42 | 74 | – |
| 17 | Diabetes Mellitus, Type 2 | 147620 | IL6 | 7p21 | 1 | 1 | 1 | 1 |
|  |  | 601487 | PPARG | 3p25 | 6 | 1 | 1 | 1 |
|  |  | 147545 | IRS1 | 2q36 | 92 | 2 | 2 | 3 |
|  |  | 600797 | IRS2 | 13q34 | 46 | 3 | 3 | 3 |
|  |  | 611259 | CDKAL1 | 6p22.3 | 18 | 13 | 7 | 3 |
|  |  | 600733 | IPF1 | 13q12.1 | 31 | 4 | 7 | 4 |
|  |  | 602228 | TCF7L2 | 10q25.3 | 88 | 5 | 7 | 4 |
|  |  | 142410 | HNF1A | 12q24.2 | 57 | 4 | 6 | 5 |
|  |  | 600281 | HNF4A | 20q12–q13.1 | 82 | 5 | 8 | 6 |
|  |  | 600937 | KCNJ11 | 11p15.1 | 26 | 14 | 15 | 7 |
|  |  | 164731 | AKT2 | 19q13.1–q13.2 | 89 | 8 | 11 | 9 |
|  |  | 138160 | SLC2A2 | 3q26.1–q26.3 | 72 | 11 | 18 | 9 |
|  |  | 189907 | HNF1B | 17q12 | 42 | 10 | 16 | 10 |
|  |  | 601724 | NEUROD1 | 2q32 | 44 | 10 | 19 | 11 |
|  |  | 605565 | RETN | 19p13.2 | 29 | 16 | 15 | 12 |
|  |  | 600509 | ABCC8 | 11p15.1 | 48 | 12 | 20 | 13 |
|  |  | 138079 | GCK | 7p15–p13 | 76 | 11 | 21 | 13 |
|  |  | 167413 | PAX4 | 7q32 | 73 | 23 | 31 | 15 |
|  |  | 604641 | MAPK8IP1 | 11p12–p11.2 | 33 | 18 | 22 | 16 |
|  |  | 605286 | CAPN10 | 2q37.3 | 29 | 42 | 39 | 16 |
|  |  | 173335 | ENPP1 | 6q22–q23 | 24 | 20 | 21 | 17 |
|  |  | 608289 | IGF2BP2 | 3q28 | 25 | 42 | 35 | 21 |
|  |  | 611145 | SLC30A8 | 8q24.11 | 23 | 32 | 33 | 23 |
|  |  | 138430 | GPD2 | 2q24.1 | 76 | 24 | 43 | 32 |
|  |  | 151670 | LIPC | 15q21–q23 | 82 | 29 | 47 | 40 |
| 18 | Esophageal Neoplasms | 190182 | TGFBR2 | 3p22 | 15 | 5 | 5 | 6 |
|  |  | 605131 | WWOX | 16q23.3–q24.1 | 19 | 3 | 7 | 7 |
|  |  | 606551 | LZTS1 | 8p22 | 44 | 22 | 35 | 30 |
|  |  | 604242 | RNF6 | 13q12.11 | 56 | 64 | 68 | 65 |
|  |  | 604050 | DLEC1 | 3p22–p21.3 | 91 | 55 | 78 | 74 |
| 19 | Glaucoma | 601771 | CYP1B1 | 2p22–p21 | 2 | 12 | 2 | 1 |
|  |  | 162662 | NTF5 | 19q13.3 | 7 | 27 | 12 | 12 |
|  |  | 602091 | LTBP2 | 14q24 | 5 | 77 | 18 | 18 |
|  |  | 602432 | OPTN | 10p15–p14 | 12 | 30 | 20 | 18 |
|  |  | 601652 | MYOC | 1q24.3–q25.2 | 41 | 20 | 22 | 20 |
|  |  | 609669 | WDR36 | 5q21.3–q22.1 | 11 | 50 | 32 | 27 |
|  |  | 605290 | OPA1 | 3q28–q29 | 78 | 49 | 65 | 55 |
| 20 | Glioma | 601728 | PTEN | 10q23.31 | 57 | 1 | 1 | – |
| 21 | Huntington Disease | 176640 | PRNP | 20pter–p12 | 9 | 10 | 10 | 10 |
|  |  | 605268 | JPH3 | 16q24.3 | 84 | 42 | 50 | 50 |
| 22 | Hyperlipidemia, Familial Combined | 609708 | LPL | 8p22 | 86 | 2 | 3 | 3 |
|  |  | 191523 | USF1 | 1q22–q23 | 89 | 6 | 11 | 11 |
| 23 | Keratosis, Actinic | 171834 | PIK3CA | 3q26.3 | 55 | 1 | 3 | 3 |
|  |  | 313020 | SAT1 | Xp22.1 | 3 | 56 | 10 | 10 |
|  |  | 125670 | DSG1 | 18q12.1–q12.2 | 6 | 37 | 12 | 10 |
|  |  | 125647 | DSP | 6p24 | 29 | 12 | 13 | 13 |
|  |  | 139350 | KRT1 | 12q13 | 68 | 33 | 45 | 43 |
| 24 | Leukemia, Myeloid, Acute | 136351 | FLT3 | 13q12 | 1 | 8 | 1 | 1 |
|  |  | 151385 | RUNX1 | 21q22.3 | 13 | 2 | 2 | 2 |
|  |  | 164040 | NPM1 | 5q35 | 11 | 3 | 4 | 5 |
|  |  | 600618 | ETV6 | 12p13 | 85 | 13 | 20 | 17 |
|  |  | 606681 | NSD1 | 5q35 | 14 | 26 | 20 | 20 |
|  |  | 604332 | CHIC2 | 4q11–q12 | 12 | 60 | 31 | 29 |
|  |  | 604763 | ARHGEF12 | 11q23.3 | 41 | 24 | 30 | 30 |
|  |  | 603025 | PICALM | 11q14 | 5 | 68 | 33 | 33 |
|  |  | 607083 | WHSC1L1 | 8p12 | 11 | 71 | 33 | 33 |
|  |  | 164690 | ABL2 | 1q24–q25 | 84 | 29 | 45 | 43 |
|  |  | 602409 | MLLT10 | 10p12 | 59 | 35 | 50 | 48 |
|  |  | 601768 | SH3GL1 | 19p13.3 | 16 | 70 | 53 | 53 |
|  |  | 600700 | LPP | 3q28 | 36 | 48 | 54 | 55 |
|  |  | 601402 | MLF1 | 3q25.1 | 56 | 48 | 62 | 58 |
|  |  | 604061 | 9–Sep | 17q25 | 56 | 57 | 72 | 71 |
| 25 | Lung Neoplasms | 131550 | EGFR | 7p12.3–p12.1 | 55 | 1 | 1 | 1 |
|  |  | 164870 | ERBB2 | 17q21.1 | 55 | 1 | 1 | 1 |
|  |  | 190070 | KRAS | 12p12.1 | 35 | 2 | 4 | 3 |
|  |  | 164757 | BRAF | 7q34 | 59 | 6 | 5 | 5 |
|  |  | 601763 | CASP8 | 2q33 | 59 | 4 | 5 | 5 |
|  |  | 605082 | RASSF1 | 3p21.3 | 21 | 6 | 10 | 10 |
|  |  | 606989 | MPO | 17q23.1 | 84 | 6 | 14 | 13 |
|  |  | 602544 | PARK2 | 6q25.2–q27 | 90 | 14 | 24 | 22 |
|  |  | 191195 | MAP3K8 | 10p11.2 | 48 | 21 | 23 | 23 |
|  |  | 609413 | ERCC6 | 10q11 | 79 | 14 | 29 | 28 |
|  |  | 603113 | PPP2R1B | 11q22–q24 | 62 | 44 | 52 | 49 |
|  |  | 602631 | SLC22A18 | 11p15.5 | 15 | 67 | 49 | 51 |
|  |  | 122720 | CYP2A6 | 19q13.2 | 90 | 34 | 56 | 53 |
|  |  | 604050 | DLEC1 | 3p22–p21.3 | 52 | 55 | 66 | 61 |
|  |  | 118503 | CHRNA3 | 15q25.1 | 99 | 53 | 75 | 72 |
|  |  | 118505 | CHRNA5 | 15q25.1 | 73 | 64 | 84 | 76 |
| 26 | Malaria | 147840 | ICAM1 | 19p13.3–p13.2 | 5 | 1 | 1 | 1 |
|  |  | 163730 | NOS2A | 17cen–q11.2 | 64 | 2 | 3 | 3 |
|  |  | 173510 | CD36 | 7q11.2 | 13 | 9 | 7 | 7 |
|  |  | 109270 | SLC4A1 | 17q21–q22 | 1 | 26 | 7 | 7 |
|  |  | 120620 | CR1 | 1q32 | 16 | 8 | 13 | 13 |
|  |  | 606252 | TIRAP | 11q23–q24 | 8 | 19 | 14 | 14 |
|  |  | 609148 | NCR3 | 6p21.3 | 19 | 9 | 15 | 14 |
|  |  | 111300 | GYPA | 4q28.2–q31.1 | 5 | 17 | 15 | 15 |
|  |  | 110750 | GYPC | 2q14–q21 | 41 | 19 | 25 | 22 |
|  |  | 604590 | FCGR2B | 1q22 | 22 | 55 | 37 | 37 |
| 27 | Melanoma | 600160 | CDKN2A | 9p21 | 13 | 2 | 3 | 2 |
|  |  | 123829 | CDK4 | 12q14 | 8 | 3 | 3 | 3 |
|  |  | 613113 | NF1 | 17q11.2 | 85 | 4 | 5 | 4 |
|  |  | 164757 | BRAF | 7q34 | 64 | 6 | 8 | 8 |
|  |  | 602150 | SNAI2 | 8q11 | 14 | 22 | 11 | 11 |
|  |  | 602216 | STK11 | 19p13.3 | 34 | 10 | 14 | 13 |
|  |  | 600675 | XRCC3 | 14q32.3 | 77 | 28 | 45 | 42 |
| 28 | Mesothelioma | 603517 | BCL10 | 1p22 | 65 | 12 | 12 | – |
| 29 | Myelodysplastic Syndromes | 604443 | ACSL6 | 5q31 | 77 | 52 | 83 | – |
| 30 | Obesity | 176830 | POMC | 2p23.3 | 73 | 1 | 3 | 1 |
|  |  | 601487 | PPARG | 3p25 | 13 | 1 | 1 | 2 |
|  |  | 164160 | LEP | 7q31.3 | 22 | 4 | 3 | 3 |
|  |  | 604630 | NR0B2 | 1p36.1 | 60 | 6 | 9 | 8 |
|  |  | 601693 | UCP2 | 11q13 | 16 | 11 | 11 | 8 |
|  |  | 109690 | ADRB2 | 5q32–q34 | 62 | 6 | 11 | 10 |
|  |  | 113730 | UCP1 | 4q31 | 37 | 11 | 12 | 10 |
|  |  | 600456 | NTRK2 | 9q22.1 | 49 | 11 | 12 | 11 |
|  |  | 155541 | MC4R | 18q22 | 33 | 21 | 20 | 11 |
|  |  | 605353 | GHRL | 3p26–p25 | 38 | 19 | 19 | 12 |
|  |  | 109691 | ADRB3 | 8p12–p11.2 | 55 | 21 | 22 | 12 |
|  |  | 602311 | AGRP | 16q22 | 64 | 28 | 27 | 13 |
|  |  | 186357 | SDC3 | 1pter–p22.3 | 95 | 15 | 18 | 14 |
|  |  | 608886 | PPARGC1B | 5q33 | 50 | 15 | 17 | 15 |
|  |  | 602044 | UCP3 | 11q13 | 62 | 28 | 26 | 18 |
|  |  | 162150 | PCSK1 | 5q15–q21 | 89 | 16 | 28 | 18 |
|  |  | 602025 | MC3R | 20q13.11–q13.2 | 38 | 27 | 31 | 19 |
|  |  | 173335 | ENPP1 | 6q22–q23 | 59 | 20 | 25 | 20 |
|  |  | 603128 | SIM1 | 6q16.3–q21 | 12 | 34 | 31 | 24 |
|  |  | 600450 | AKR1C2 | 10p15–p14 | 9 | 37 | 29 | 30 |
|  |  | 600781 | PYY | 17q21 | 72 | 42 | 49 | 41 |
|  |  | 602606 | CARTPT | 5q13.2 | 97 | 45 | 64 | 44 |
|  |  | 300444 | SLC6A14 | Xq23–q24 | 35 | 61 | 66 | 67 |
| 31 | Osteoarthritis | 155760 | ACAN | 15q26.1 | 91 | 7 | 14 | 13 |
|  |  | 605083 | FRZB | 2q31–q33 | 58 | 44 | 47 | 44 |
|  |  | 608135 | ASPN | 9q21.3–q22 | 82 | 42 | 62 | 57 |
|  |  | 602109 | MATN3 | 2p24–p23 | 86 | 52 | 75 | 72 |
| 32 | Paraplegia | 118190 | HSPD1 | 2q33.1 | 22 | 10 | 10 | 9 |
|  |  | 602783 | SPG7 | 16q24.3 | 27 | 15 | 10 | 9 |
|  |  | 603690 | SLC33A1 | 3q25.31 | 53 | 17 | 24 | 24 |
|  |  | 610844 | SPG11 | 15q21.1 | 8 | 70 | 30 | 26 |
|  |  | 609139 | REEP1 | 2p11.2 | 16 | 69 | 43 | 29 |
|  |  | 603197 | PNPLA6 | 19p13.3 | 10 | 62 | 35 | 31 |
|  |  | 300401 | PLP1 | Xq22 | 75 | 24 | 38 | 33 |
|  |  | 602821 | KIF5A | 12q13 | 24 | 45 | 47 | 36 |
|  |  | 608145 | NIPA1 | 15q11.1 | 30 | 56 | 48 | 36 |
|  |  | 606439 | SPG3A | 14q11–q21 | 34 | 53 | 50 | 37 |
|  |  | 604277 | SPAST | 2p22–p21 | 35 | 47 | 52 | 45 |
|  |  | 610657 | KIAA0196 | 8q24.13 | 28 | 68 | 55 | 47 |
|  |  | 610243 | ZFYVE27 | 10q24.2 | 31 | 77 | 68 | 54 |
|  |  | 612319 | FA2H | 16q21–q23.1 | 28 | 75 | 64 | 64 |
|  |  | 603711 | CYP7B1 | 8q21.3 | 94 | 44 | 68 | 67 |
|  |  | 608803 | GJC2 | 1q41–q42 | 83 | 72 | 88 | 86 |
|  |  | 612012 | ZFYVE26 | 14q24.1 | 74 | 81 | 94 | 90 |
| 33 | Parkinson Disease | 600075 | TBP | 6q27 | 71 | 2 | 3 | 4 |
|  |  | 157140 | MAPT | 17q21.1 | 36 | 6 | 6 | 6 |
|  |  | 163890 | SNCA | 4q21 | 1 | 14 | 8 | 7 |
|  |  | 602533 | PARK7 | 1p36 | 25 | 9 | 11 | 8 |
|  |  | 602544 | PARK2 | 6q25.2–q27 | 52 | 14 | 17 | 14 |
|  |  | 606441 | HTRA2 | 2p12 | 39 | 16 | 20 | 14 |
|  |  | 605648 | FBXO7 | 22q12–q13 | 2 | 40 | 19 | 19 |
|  |  | 610513 | ATP13A2 | 1p36 | 17 | 64 | 46 | 43 |
|  |  | 608309 | PINK1 | 1p36 | 42 | 52 | 54 | 45 |
|  |  | 612003 | TNRC15 | 2q37.1 | 78 | 77 | 84 | 83 |
| 34 | Polycystic Ovary Syndrome | 136470 | FST | 5q11.2 | 29 | 5 | 7 | 7 |
|  |  | 313700 | AR | Xq11–q12 | 1 | 1 | 1 | 1 |
|  |  | 157145 | MSMB | 10q11.2 | 1 | 22 | 2 | 2 |
| 35 | Prostatic Neoplasms | 601728 | PTEN | 10q23.31 | 87 | 1 | 2 | 2 |
|  |  | 600623 | CD82 | 11p11.2 | 9 | 8 | 8 | 4 |
|  |  | 600185 | BRCA2 | 13q12.3 | 85 | 6 | 8 | 5 |
|  |  | 604373 | CHEK2 | 22q12.1 | 84 | 4 | 10 | 7 |
|  |  | 600997 | EPHB2 | 1p36.1–p35 | 89 | 6 | 17 | 11 |
|  |  | 611955 | HNF1B | 17q12 | 50 | 10 | 16 | 12 |
|  |  | 180435 | RNASEL | 1q25 | 8 | 36 | 27 | 15 |
|  |  | 600020 | MXI1 | 10q25 | 29 | 22 | 23 | 21 |
|  |  | 104155 | ZFHX3 | 16q22.3–q23.1 | 20 | 34 | 31 | 21 |
|  |  | 601767 | HIP1 | 7q11.23 | 67 | 28 | 28 | 26 |
|  |  | 602686 | MAD1L1 | 7p22 | 74 | 24 | 38 | 27 |
|  |  | 609922 | EHBP1 | 2p15 | 20 | 54 | 37 | 30 |
|  |  | 605367 | ELAC2 | 17p11 | 92 | 42 | 64 | 36 |
| 36 | Sarcoma | 600192 | SS18 | 18q11.2 | 28 | 53 | 46 | 46 |
|  |  | 300192 | SSX2 | Xp11.2 | 26 | 70 | 55 | 56 |
|  |  | 300326 | SSX4 | Xp11.2 | 34 | 73 | 59 | 59 |
| 37 | Schizophrenia | 164730 | AKT1 | 14q32.3 | 55 | 1 | 1 | 1 |
|  |  | 607093 | MTHFR | 1p36.3 | 88 | 2 | 12 | 8 |
|  |  | 601525 | CHI3L1 | 1q32.1 | 3 | 23 | 9 | 9 |
|  |  | 116790 | COMT | 22q11.2 | 23 | 7 | 11 | 9 |
|  |  | 607145 | DTNBP1 | 6p22.3 | 24 | 20 | 19 | 10 |
|  |  | 118511 | CHRNA7 | 15q14 | 26 | 24 | 25 | 19 |
|  |  | 126451 | DRD3 | 3q13.3 | 95 | 20 | 27 | 19 |
|  |  | 605566 | RTN4R | 22q11 | 25 | 31 | 26 | 20 |
|  |  | 182135 | HTR2A | 13q14–q21 | 44 | 21 | 29 | 21 |
|  |  | 605210 | DISC1 | 1q42.1 | 90 | 20 | 32 | 22 |
|  |  | 600755 | SYN2 | 3p25 | 9 | 39 | 26 | 23 |
|  |  | 606810 | PRODH | 22q11.2 | 46 | 29 | 32 | 23 |
|  |  | 124050 | DAO | 12q24 | 13 | 59 | 45 | 34 |
|  |  | 607254 | APOL4 | 22q12.3 | 31 | 64 | 56 | 42 |
|  |  | 607252 | APOL2 | 22q12.3 | 30 | 75 | 65 | 60 |
|  |  | 606666 | LGR4 | 11p14–p13 | 27 | 81 | 73 | 73 |
| 38 | Thrombocythemia, Essential | 147796 | JAK2 | 9p24 | 7 | 1 | 1 | 1 |
|  |  | 159530 | MPL | 1p34 | 5 | 10 | 4 | 4 |
|  |  | 600044 | THPO | 3q26.3–q27 | 67 | 14 | 14 | 12 |
| 39 | Thyroid Neoplasms | 190020 | HRAS | 11p15.5 | 29 | 4 | 4 | 4 |
|  |  | 188830 | PRKAR1A | 17q23–q24 | 37 | 4 | 8 | 8 |
|  |  | 603372 | TSHR | 14q31 | 14 | 11 | 9 | 9 |
|  |  | 601984 | NCOA4 | 10q11.2 | 32 | 19 | 19 | 16 |
|  |  | 603406 | TRIM24 | 7q32–q34 | 51 | 16 | 23 | 17 |
|  |  | 609435 | NDUFA13 | 19p13.2–p13.1 | 24 | 34 | 30 | 30 |
|  |  | 601985 | CCDC6 | 10q21 | 38 | 37 | 37 | 33 |
|  |  | 606918 | GOLGA5 | 14q | 34 | 46 | 44 | 35 |
|  |  | 605769 | TRIM33 | 1p13 | 57 | 52 | 56 | 43 |
|  |  | 605391 | MINPP1 | 10q23 | 23 | 61 | 44 | 46 |
|  |  | 600299 | PCM1 | 8p22–p21.3 | 60 | 33 | 55 | 51 |
| 40 | Urinary Bladder Neoplasms | 134934 | FGFR3 | 4p16.3 | 17 | 1 | 1 | 1 |
|  |  | 180200 | RB1 | 13q14.1–q14.2 | 76 | 1 | 1 | 1 |
|  |  | 190070 | KRAS | 12p12.1 | 13 | 2 | 2 | 2 |
|  |  | 190020 | HRAS | 11p15.5 | 16 | 4 | 4 | 4 |
