# Supplementary material for: Ranking Candidate Disease Genes from Gene Expression and Protein Interaction: A Katz-Centrality Based Approach
Source: PLoS One. 2011 Sep 2;6(9):e24306. doi: 10.1371/journal.pone.0024306 (PMC3166320; doi:10.1371/journal.pone.0024306)
Supplement: Table S3 — Unidentified genes on known chromosomal regions associated with the diseases under study, from OMIM morbid map. (DOCX) [file pone.0024306.s003.docx]

| Disease MeSH | OMIM ID | Gene Symbol in OMIM | Gene Loci |
| --- | --- | --- | --- |
| Alzheimer Disease | 609636 | AD10 | 7q36 |
|  | 609790 | AD11 | 9p22.1 |
|  | 611073 | AD12 | 8p12–q22 |
|  | 611152 | AD13 | 1q21 |
|  | 611154 | AD14 | 1q25 |
|  | 611155 | AD15 | 3q22–q24 |
|  | 300756 | AD16 | Xq21.3 |
|  | 602096 | AD5 | 12p11.23–q13.12 |
|  | 605526 | AD6 | 10q24 |
|  | 606187 | AD7 | 10p13 |
|  | 607116 | AD8 | 20p |
|  | 608907 | AD9 | 19p13.2 |
| Amyotrophic Lateral Sclerosis | 606640 | ALS3 | 18q21 |
|  | 602099 | ALS5 | 15q15.1–q21.1 |
|  | 608031 | ALS7 | 20p13 |
|  | 105550 | ALSFTD1 | 9q21–q22 |
| Asthma | 609958 | ASRT3 | 2p16 |
|  | 610906 | ASRT4 | 1p31 |
|  | 611403 | ASRT6 | 17q21 |
|  | 613207 | ASRT8 | 9q33 |
| Carcinoma, Renal Cell | 605075 | RCCP3 | 17q21.32 |
| Cardiomyopathy | 600884 | CMD1B | 9q13 |
|  | 601493 | CMD1C | 10q21–q23 |
|  | 602067 | CMD1F | 6q23 |
|  | 604288 | CMD1H | 2q14–q22 |
|  | 605582 | CMD1K | 6q12–q16 |
|  | 609915 | CMD1Q | 7q22.3–q31.1 |
|  | 609578 | RCM2 | 10q23.3 |
| Colorectal Neoplasms | 612591 | CRCS10 | 19q13.1 |
|  | 612592 | CRCS11 | 20p12.3 |
|  | 611469 | CRCS2 | 8q24 |
|  | 612230 | CRCS5 | 10p14 |
|  | 612231 | CRCS6 | 8q23 |
|  | 612232 | CRCS7 | 11q23 |
|  | 612589 | CRCS8 | 14q22.2 |
|  | 601228 | CRCS4 | 15q15.3–q22.1 |
| Depression | 608520 | MDD1 | 12q22–q23.2 |
|  | 608691 | MDD2 | 15q25.3–q26.2 |
| Diabetes Mellitus, Type 2 | 601407 | NIDDM2 | 12q24.2 |
|  | 603694 | NIDDM3 | 20q12–q13.1 |
|  | 608036 | NIDDM4 | 5q34–q35.2 |
| Glaucoma | 606689 | GLC1B | 2cen–q13 |
|  | 601682 | GLC1C | 3q21–q24 |
|  | 602429 | GLC1D | 8q23 |
|  | 603383 | GLC1F | 7q35–q36 |
|  | 609745 | GLC1I | 15q11–q13 |
|  | 608696 | GLC1K | 20p12 |
|  | 610535 | GLC1M | 5q22.1–q32 |
|  | 600975 | GLC3B | 1p36.2–p36.1 |
|  | 613085 | GLC3C | 14q24.3 |
|  | 608695 | JOAG2 | 9q22 |
| Glioma | 607248 | GLM4 | 15q23–q26.3 |
|  | 613030 | GLM5 | 9p21.3 |
|  | 613031 | GLM6 | 20q13.33 |
|  | 613033 | GLM8 | 5p15.33 |
| Huntington Disease | 604802 | HDL3 | 4p15.3 |
| Hyperlipidemia, Familial Combined | 604499 | HYPLIP2 | 11p |
| Lung Neoplasms | 608935 | LNCR1 | 6q23–q25 |
|  | 612571 | LNCR3 | 5p15.33 |
|  | 612593 | LNCR4 | 6p21.33 |
| Malaria | 248310 | PFBI | 5q31–q33 |
| Melanoma | 155600 | CMM | 1p36 |
|  | 608035 | CMM4 | 1p22 |
|  | 612263 | CMM7 | 20q11.2 |
|  | 606661 | UBM2 | 3p25.2–p25.1 |
|  | 606660 | UVM1 | 3q24–q26 |
| Mesothelioma | 156240 | MMS | 9p |
| Obesity | 607514 | BMIQ10 | 10q |
|  | 608410 | BMIQ7 | 4p15–p14 |
|  | 603188 | BMIQ8 | 10p |
| Osteoarthritis | 610839 | OS4 | 2q33.3 |
|  | 612401 | OS6 | 3p24.3 |
| Paraplegia | 604805 | SPG12 | 19q13 |
|  | 605229 | SPG14 | 3q27–q28 |
|  | 300266 | SPG16 | Xq11.2 |
|  | 611225 | SPG18 | 8p12–p11.21 |
|  | 607152 | SPG19 | 9q |
|  | 607584 | SPG24 | 13q14 |
|  | 608220 | SPG25 | 6q23–q24.1 |
|  | 609195 | SPG26 | 12p11.1–q14 |
|  | 609041 | SPG27 | 10q22.1–q24.1 |
|  | 609340 | SPG28 | 14q21.3–q22.3 |
|  | 609727 | SPG29 | 1p31.1–p21.1 |
|  | 611252 | SPG32 | 14q12–q21 |
|  | 300750 | SPG34 | Xq24–q25 |
|  | 613096 | SPG36 | 12q23–q24 |
|  | 611945 | SPG37 | 8p21.1–q13.3 |
|  | 612335 | SPG38 | 4p16–p15 |
|  | 613364 | SPG41 | 11p14.1–p11.2 |
|  | 613162 | SPG45 | 10q24.3–q25.1 |
|  | 601162 | SPG9 | 10q23.3–q24.1 |
|  | 609541 | SPOAN | 11q13 |
| Parkinson Disease | 606852 | PARK10 | 1p32 |
|  | 300557 | PARK12 | Xq21–q25 |
|  | 613164 | PARK16 | 1q32 |
|  | 602404 | PARK3 | 2p13 |
| Prostatic Neoplasms | 611100 | HPC10 | 8q24 |
|  | 611958 | HPC14 | 11q13 |
|  | 611959 | HPC15 | 19q13.4 |
|  | 608656 | HPC3 | 20q13 |
|  | 608658 | HPC4 | 7p11–q21 |
|  | 609299 | HPC5 | 3p26 |
|  | 609558 | HPC6 | 22q12.3 |
|  | 610321 | HPC7 | 15q12 |
|  | 610997 | HPC9 | 17q21–q22 |
|  | 607592 | HPCQTL19 | 19q |
|  | 300147 | HPCX1 | Xq27–q28 |
|  | 300704 | HPCX2 | Xp11.22 |
|  | 602759 | PCAP | 1q42.2–q43 |
| Schizophrenia | 181510 | SCZD1 | 5q23–q35 |
|  | 605419 | SCZD10 | 15q15 |
|  | 608078 | SCZD11 | 10q22.3 |
|  | 608543 | SCZD12 | 1p36.2 |
|  | 613025 | SCZD13 | 15q13 |
|  | 612361 | SCZD14 | 2q32.1 |
|  | 603342 | SCZD2 | 11q14–q21 |
|  | 600511 | SCZD3 | 6p23 |
|  | 603013 | SCZD6 | 8p21 |
|  | 603176 | SCZD7 | 13q32 |
|  | 603206 | SCZD8 | 18p |
| Thyroid Neoplasms | 606240 | NMTC1 | 2q21 |
|  | 605642 | PRN1 | 1q21 |
|  | 603386 | TCO | 19p13.2 |
