# Supplementary material for: Ranking Candidate Disease Genes from Gene Expression and Protein Interaction: A Katz-Centrality Based Approach
Source: PLoS One. 2011 Sep 2;6(9):e24306. doi: 10.1371/journal.pone.0024306 (PMC3166320; doi:10.1371/journal.pone.0024306)
Supplement: Table S4 — List of the top ranked genes, i.e., the top 200 s 1-ranked genes in over 90% diseases under study. (DOCX) [file pone.0024306.s004.docx]

| No | Gene ID | Gene symbol | Disease gene under study | Percentage of s–rank in top 200 |
| --- | --- | --- | --- | --- |
| 1 | 207 | AKT1 | 1 | 0.976 |
| 2 | 7157 | TP53 | 1 | 0.976 |
| 11 | 3569 | IL6 | 1 | 0.976 |
| 12 | 595 | CCND1 | 1 | 0.976 |
| 14 | 1956 | EGFR | 1 | 0.976 |
| 16 | 3265 | HRAS | 1 | 0.976 |
| 18 | 5290 | PIK3CA | 1 | 0.976 |
| 19 | 5468 | PPARG | 1 | 0.976 |
| 20 | 5728 | PTEN | 1 | 0.976 |
| 22 | 367 | AR | 1 | 0.976 |
| 23 | 581 | BAX | 1 | 0.976 |
| 24 | 1499 | CTNNB1 | 1 | 0.976 |
| 26 | 2033 | EP300 | 1 | 0.976 |
| 27 | 2064 | ERBB2 | 1 | 0.976 |
| 29 | 3383 | ICAM1 | 1 | 0.976 |
| 31 | 3667 | IRS1 | 1 | 0.976 |
| 32 | 3717 | JAK2 | 1 | 0.976 |
| 34 | 4843 | NOS2A | 1 | 0.976 |
| 35 | 5925 | RB1 | 1 | 0.976 |
| 37 | 7099 | TLR4 | 1 | 0.976 |
| 3 | 3481 | IGF2 | 0 | 0.976 |
| 4 | 723961 | INS–IGF2 | 0 | 0.976 |
| 5 | 5595 | MAPK3 | 0 | 0.976 |
| 6 | 6714 | SRC | 0 | 0.976 |
| 7 | 3630 | INS | 0 | 0.976 |
| 8 | 5599 | MAPK8 | 0 | 0.976 |
| 9 | 2353 | FOS | 0 | 0.976 |
| 10 | 4609 | MYC | 0 | 0.976 |
| 13 | 3553 | IL1B | 0 | 0.976 |
| 15 | 2335 | FN1 | 0 | 0.976 |
| 17 | 3576 | IL8 | 0 | 0.976 |
| 21 | 6772 | STAT1 | 0 | 0.976 |
| 25 | 1950 | EGF | 0 | 0.976 |
| 28 | 3320 | HSP90AA1 | 0 | 0.976 |
| 30 | 3458 | IFNG | 0 | 0.976 |
| 33 | 4318 | MMP9 | 0 | 0.976 |
| 36 | 6774 | STAT3 | 0 | 0.976 |
| 38 | 356 | FASLG | 0 | 0.976 |
| 39 | 596 | BCL2 | 0 | 0.976 |
| 40 | 598 | BCL2L1 | 0 | 0.976 |
| 41 | 808 | CALM3 | 0 | 0.976 |
| 42 | 836 | CASP3 | 0 | 0.976 |
| 43 | 920 | CD4 | 0 | 0.976 |
| 44 | 925 | CD8A | 0 | 0.976 |
| 45 | 960 | CD44 | 0 | 0.976 |
| 46 | 998 | CDC42 | 0 | 0.976 |
| 47 | 999 | CDH1 | 0 | 0.976 |
| 48 | 1027 | CDKN1B | 0 | 0.976 |
| 49 | 1277 | COL1A1 | 0 | 0.976 |
| 50 | 1385 | CREB1 | 0 | 0.976 |
| 51 | 1432 | MAPK14 | 0 | 0.976 |
| 52 | 1437 | CSF2 | 0 | 0.976 |
| 53 | 1958 | EGR1 | 0 | 0.976 |
| 54 | 2099 | ESR1 | 0 | 0.976 |
| 55 | 2534 | FYN | 0 | 0.976 |
| 56 | 2885 | GRB2 | 0 | 0.976 |
| 57 | 2908 | NR3C1 | 0 | 0.976 |
| 58 | 2932 | GSK3B | 0 | 0.976 |
| 59 | 3065 | HDAC1 | 0 | 0.976 |
| 60 | 3091 | HIF1A | 0 | 0.976 |
| 61 | 3308 | HSPA4 | 0 | 0.976 |
| 62 | 3479 | IGF1 | 0 | 0.976 |
| 63 | 3558 | IL2 | 0 | 0.976 |
| 64 | 3565 | IL4 | 0 | 0.976 |
| 65 | 3643 | INSR | 0 | 0.976 |
| 66 | 3688 | ITGB1 | 0 | 0.976 |
| 67 | 4087 | SMAD2 | 0 | 0.976 |
| 68 | 4088 | SMAD3 | 0 | 0.976 |
| 69 | 4313 | MMP2 | 0 | 0.976 |
| 70 | 4790 | NFKB1 | 0 | 0.976 |
| 71 | 4792 | NFKBIA | 0 | 0.976 |
| 72 | 4803 | NGFB | 0 | 0.976 |
| 73 | 5111 | PCNA | 0 | 0.976 |
| 74 | 5155 | PDGFB | 0 | 0.976 |
| 75 | 5295 | PIK3R1 | 0 | 0.976 |
| 76 | 5578 | PRKCA | 0 | 0.976 |
| 77 | 5594 | MAPK1 | 0 | 0.976 |
| 78 | 5604 | MAP2K1 | 0 | 0.976 |
| 79 | 5743 | PTGS2 | 0 | 0.976 |
| 80 | 5747 | PTK2 | 0 | 0.976 |
| 81 | 5781 | PTPN11 | 0 | 0.976 |
| 82 | 5894 | RAF1 | 0 | 0.976 |
| 83 | 5970 | RELA | 0 | 0.976 |
| 84 | 6464 | SHC1 | 0 | 0.976 |
| 85 | 7040 | TGFB1 | 0 | 0.976 |
| 86 | 7132 | TNFRSF1A | 0 | 0.976 |
| 87 | 7316 | UBC | 0 | 0.976 |
| 88 | 54205 | CYCS | 0 | 0.976 |
| 89 | 355 | FAS | 1 | 0.951 |
| 90 | 5328 | PLAU | 1 | 0.951 |
| 91 | 6347 | CCL2 | 1 | 0.951 |
| 92 | 7852 | CXCR4 | 0 | 0.951 |
| 93 | 2130 | EWSR1 | 0 | 0.951 |
| 94 | 2247 | FGF2 | 0 | 0.951 |
| 95 | 3552 | IL1A | 0 | 0.951 |
| 96 | 5294 | PIK3CG | 0 | 0.951 |
| 97 | 672 | BRCA1 | 0 | 0.927 |
| 98 | 857 | CAV1 | 0 | 0.927 |
| 99 | 2308 | FOXO1 | 0 | 0.927 |
| 100 | 4089 | SMAD4 | 0 | 0.927 |
| 101 | 5159 | PDGFRB | 0 | 0.927 |
| 102 | 5601 | MAPK9 | 0 | 0.927 |
| 103 | 6387 | CXCL12 | 0 | 0.927 |
| 104 | 7431 | VIM | 0 | 0.927 |
| 105 | 8874 | ARHGEF7 | 0 | 0.927 |
| 106 | 3932 | LCK | 0 | 0.902 |
| 107 | 7042 | TGFB2 | 0 | 0.902 |
