# Supplementary material for: Ranking Candidate Disease Genes from Gene Expression and Protein Interaction: A Katz-Centrality Based Approach
Source: PLoS One. 2011 Sep 2;6(9):e24306. doi: 10.1371/journal.pone.0024306 (PMC3166320; doi:10.1371/journal.pone.0024306)
Supplement: Table S5 — Disease pathways significantly enriched with the top ranked genes. (DOCX) [file pone.0024306.s005.docx]

| Disease category | KEGG disease pathway name | Disease MeSH term | Mapped genes | Total genes |
| --- | --- | --- | --- | --- |
| Cancers | Renal cell carcinoma | *Carcinoma, Renal Cell | 17 | 70 |
|  | Colorectal cancer | *Colorectal Neoplasms | 25 | 62 |
|  | Glioma | *Glioma | 22 | 65 |
|  | Acute myeloid leukemia | *Leukemia, Myeloid, Acute | 15 | 58 |
|  | Small cell lung cancer | *Lung Neoplasms | 21 | 85 |
|  | Non–small cell lung cancer | *Lung Neoplasms | 17 | 54 |
|  | Melanoma | *Melanoma | 20 | 71 |
|  | Prostate cancer | *Prostatic Neoplasms | 33 | 89 |
|  | Thyroid cancer | *Thyroid Neoplasms | 10 | 29 |
|  | Bladder cancer | *Urinary Bladder Neoplasms | 16 | 42 |
|  | Endometrial cancer | Endometrial Neoplasms | 20 | 52 |
|  | Chronic myeloid leukemia | Leukemia, Myeloid, Chronic | 26 | 73 |
|  | Pathways in cancer(overview) | Neoplasms | 64 | 327 |
|  | Pancreatic cancer | Pancreatic Neoplasms | 27 | 70 |
| Cardiovascular Diseases | Hypertrophic cardiomyopathy (HCM) | *Cardiomyopathy | 5 | 87 |
|  | Viral myocarditis | – | 6 | 72 |
| Immune System Diseases | Primary immunodeficiency | – | 3 | 35 |
|  | Autoimmune thyroid disease | – | 4 | 54 |
|  | Allograft rejection | – | 5 | 39 |
|  | Graft–versus–host disease | Graft vs Host Disease | 7 | 43 |
|  | Rheumatoid arthritis | *Arthritis, Rheumatoid | 13 | 92 |
| Infectious Diseases | Pathogenic Escherichia coli infection | – | 7 | 57 |
|  | African trypanosomiasis | Trypanosomiasis, African | 7 | 35 |
|  | Malaria | *Malaria | 9 | 51 |
|  | Bacterial invasion of epithelial cells | – | 12 | 71 |
|  | Epithelial cell signaling in Helicobacter pylori infection | – | 12 | 68 |
|  | Shigellosis | Dysentery, Bacillary | 12 | 62 |
|  | Hepatitis C | Hepatitis C | 23 | 134 |
|  | Measles | Measles | 24 | 134 |
|  | Tuberculosis | Tuberculosis | 28 | 180 |
|  | Amoebiasis | Amoebiasis | 19 | 106 |
|  | Toxoplasmosis | Toxoplasmosis | 26 | 133 |
|  | Influenza A | Influenza A | 31 | 176 |
|  | Leishmaniasis | Leishmaniasis | 19 | 73 |
|  | Chagas disease (American trypanosomiasis) | Chagas Disease | 28 | 104 |
|  | Pertussis | Whooping Cough | 17 | 74 |
| Metabolic Diseases | Type I diabetes mellitus | Diabetes Mellitus, Type 1 | 7 | 45 |
|  | Type II diabetes mellitus | *Diabetes Mellitus, Type 2 | 10 | 48 |
| Neurodegenerative Diseases | Huntington’s disease | *Huntington Disease | 8 | 183 |
|  | Alzheimer’s disease | *Alzheimer Disease | 9 | 167 |
|  | Amyotrophic lateral sclerosis (ALS) | *Amyotrophic Lateral Sclerosis | 8 | 53 |
|  | Prion diseases | Prion Diseases | 9 | 35 |
