# Supplementary material for: Ranking Candidate Disease Genes from Gene Expression and Protein Interaction: A Katz-Centrality Based Approach
Source: PLoS One. 2011 Sep 2;6(9):e24306. doi: 10.1371/journal.pone.0024306 (PMC3166320; doi:10.1371/journal.pone.0024306)
Supplement: Table S6 — Pathways significantly enriched with the top ranked genes. (DOCX) [file pone.0024306.s006.docx]

| Pathway 1st level category | Pathway 2nd level category | Pathway name | Mapped genes | Total genes |
| --- | --- | --- | --- | --- |
| Cellular Processes | Cell Communication | Tight junction | 7 | 132 |
|  |  | Gap junction | 12 | 90 |
|  |  | Adherens junction | 14 | 73 |
|  |  | Focal adhesion | 33 | 200 |
|  | Cell Growth and Death | Oocyte meiosis | 7 | 114 |
|  |  | p53 signaling pathway | 8 | 69 |
|  |  | Cell cycle | 14 | 128 |
|  |  | Apoptosis | 19 | 86 |
|  | Cell Motility | Regulation of actin cytoskeleton | 19 | 214 |
|  | Transport and Catabolism | Endocytosis | 11 | 204 |
| Environmental Information Processing | Signal Transduction | Phosphatidylinositol signaling system | 6 | 78 |
|  |  | Wnt signaling pathway | 12 | 151 |
|  |  | TGF–beta signaling pathway | 10 | 85 |
|  |  | mTOR signaling pathway | 9 | 52 |
|  |  | Jak–STAT signaling pathway | 18 | 155 |
|  |  | VEGF signaling pathway | 15 | 76 |
|  |  | ErbB signaling pathway | 22 | 87 |
|  |  | MAPK signaling pathway | 31 | 272 |
|  | Signaling Molecules and Interaction | Cytokine–cytokine receptor interaction | 20 | 275 |
| Organismal Systems | Circulatory System | Vascular smooth muscle contraction | 6 | 126 |
|  | Development | Axon guidance | 10 | 130 |
|  |  | Dorso–ventral axis formation | 5 | 25 |
|  |  | Osteoclast differentiation | 26 | 128 |
|  | Digestive System | Carbohydrate digestion and absorption | 4 | 44 |
|  | Endocrine System | Melanogenesis | 11 | 101 |
|  |  | Adipocytokine signaling pathway | 11 | 69 |
|  |  | Progesterone–mediated oocyte maturation | 14 | 87 |
|  |  | GnRH signaling pathway | 15 | 101 |
|  |  | Insulin signaling pathway | 19 | 138 |
|  | Excretory System | Aldosterone–regulated sodium reabsorption | 10 | 42 |
|  | Immune System | Cytosolic DNA–sensing pathway | 5 | 62 |
|  |  | Antigen processing and presentation | 6 | 78 |
|  |  | RIG–I–like receptor signaling pathway | 7 | 71 |
|  |  | Intestinal immune network for IgA production | 6 | 49 |
|  |  | Hematopoietic cell lineage | 8 | 88 |
|  |  | Fc gamma R–mediated phagocytosis | 10 | 95 |
|  |  | Leukocyte transendothelial migration | 15 | 116 |
|  |  | NOD–like receptor signaling pathway | 13 | 59 |
|  |  | B cell receptor signaling pathway | 15 | 75 |
|  |  | Chemokine signaling pathway | 24 | 189 |
|  |  | Toll–like receptor signaling pathway | 19 | 102 |
|  |  | T cell receptor signaling pathway | 26 | 108 |
|  |  | Natural killer cell mediated cytotoxicity | 20 | 141 |
|  |  | Fc epsilon RI signaling pathway | 17 | 79 |
|  | Nervous System | Long–term depression | 7 | 70 |
|  |  | Long–term potentiation | 8 | 70 |
|  |  | Cholinergic synapse | 14 | 112 |
|  |  | Neurotrophin signaling pathway | 27 | 127 |
